# 3D Printing of Nacre-Inspired Structures with Exceptional Mechanical and Flame-Retardant Properties

**DOI:** 10.34133/2022/9840574

**Published:** 2022-01-27

**Authors:** Yang Yang, Ziyu Wang, Qingqing He, Xiangjia Li, Gengxi Lu, Laiming Jiang, Yushun Zeng, Brandon Bethers, Jie Jin, Shuang Lin, Siqi Xiao, Yizhen Zhu, Xianke Wu, Wenwu Xu, Qiming Wang, Yong Chen

**Affiliations:** ^1^Department of Mechanical Engineering, San Diego State University, 5500 Campanile Drive, San Diego, CA 92182, USA; ^2^The Institute of Technological Sciences, Wuhan University, Wuhan 430072, China; ^3^School for Engineering of Matter, Transport and Energy, Arizona State University, 551 E Tyler Mall, Tempe, AZ 85281, USA; ^4^Department of Biomedical Engineering, University of Southern California, 1042 Downey Way, Los Angeles, CA 90089, USA; ^5^Epstein Department of Industrial and Systems Engineering, University of Southern California, 3715 McClintock Ave, Los Angeles, CA 90089, USA; ^6^ShadeCraft Robotics Inc., Pasadena, CA 91105, USA; ^7^Department of Chemical Engineering and Materials Science, University of Southern California, 925 Bloom Walk, Los Angeles, California 90089, USA; ^8^Department of Aerospace and Mechanical Engineering, University of Southern California, Los Angeles, CA 90089, USA; ^9^School of Physics and Technology, Wuhan University, Wuhan 430072, China; ^10^Sonny Astani Department of Civil and Environmental Engineering, University of Southern California, Los Angeles, CA 90089, USA

## Abstract

Flame-retardant and thermal management structures have attracted great attention due to the requirement of high-temperature exposure in industrial, aerospace, and thermal power fields, but the development of protective fire-retardant structures with complex shapes to fit arbitrary surfaces is still challenging. Herein, we reported a rotation-blade casting-assisted 3D printing process to fabricate nacre-inspired structures with exceptional mechanical and flame-retardant properties, and the related fundamental mechanisms are studied. 3-(Trimethoxysilyl)propyl methacrylate (TMSPMA) modified boron nitride nanoplatelets (BNs) were aligned by rotation-blade casting during the 3D printing process to build the “brick and mortar” architecture. The 3D printed structures are more lightweight, while having higher fracture toughness than the natural nacre, which is attributed to the crack deflection, aligned BN (a-BNs) bridging, and pull-outs reinforced structures by the covalent bonding between TMSPMA grafted a-BNs and polymer matrix. Thermal conductivity is enhanced by 25.5 times compared with pure polymer and 5.8 times of anisotropy due to the interconnection of a-BNs. 3D printed heat-exchange structures with vertically aligned BNs in complex shapes were demonstrated for efficient thermal control of high-power light-emitting diodes. 3D printed helmet and armor with a-BNs show exceptional mechanical and fire-retardant properties, demonstrating integrated mechanical and thermal protection.

## 1. Introduction

Lightweight and strong flame-retardant structures have been widely studied due to the need for high-performance thermal insulation in fire-fighting, military, industrial, and aerospace engineering [1, 2]. There is also an increasing demand for thermal control structures in electronic devices due to the thermal accumulation problems in the miniaturization and multifunctional integration of electronic devices [3, 4]. Ceramics and polymers are great candidates for thermal-control/flame-retardant structures due to their good processibility and excellent mechanical, thermal, and electrical properties. However, flaw-tolerance and high-strength properties are not well combined in these two kinds of materials. There is a way in nature to generate strong and flaw tolerant structures by assembling ceramic platelets with biopolymer interlayers in nacre [5]. Nacre possesses outstanding strength, stiffness, and toughness after millions of years of evolution [6]. The well-aligned calcium carbonate nanoplatelets (95 vol%) working as “brick” and *β*-chitin and silk fibroin proteins (5 vol%) interlayers as “mortar” form the “brick-and-mortar” (BM) structure and contribute to the excellent mechanical property of natural nacre [7]. The BM structure sheds light on tough ceramic/polymer composite structure design for thermal control and flame-retardant applications. As a ceramic filler, hexagonal boron nitride nanoplatelets (BNs) possess a flaky structure with exceptional mechanical, thermal, and flame stability, which make it an attractive option for applications in body armor, heat sinks, microcircuit packaging, and fire-retardant structures [8]. Several studies have been performed on the nacre-inspired structures (e.g., ice-templated [9] and layer-by-layer self-assembly [10, 11]) and structures with aligned BNs (a-BNs) for anisotropic thermal conductivity and flame-retardant properties [3, 12, 13], but these structures are limited to thin films or simple bulk shapes with respective mechanical or thermal properties. It remains a challenge to achieve multifunctional arbitrary three-dimensional (3D) structures. Electric/magnetic field/extrusion-based 3D printing methods have been utilized to fabricate arbitrary shapes of nacre-inspired structures; however, the alignment is limited in low loading of fillers and low viscosity composites [14–18]. A high volume ratio of ceramic platelets is required for excellent mechanical property as in natural nacre. Accurate control of nacre-inspired alignment in 3D printing using highly viscous composites to fabricate high-performance multifunctional structures with arbitrary shapes is challenging.

Here, we report a rotation-blade casting-assisted 3D (*rbc*-3D) printing process to construct the nacre-inspired structure with reinforced mechanical properties, highly anisotropic thermal conductivity, and excellent flame-retardancy. The alignment mechanism of BN in high viscous slurry was first studied. The 3D printed nacre-inspired structure has a high loading of a-BNs (55 wt%) and possesses enhanced integrated mechanical/thermal/flame-retardant properties. The fracture toughness of 3D printed structures with a-BNs is higher than that in natural nacre. The mechanical and flame-retardant reinforcement mechanisms were analyzed. We further demonstrate that a 3D printed heat sink with a branching structure shows efficient thermal control and heat dissipation for high-power light-emitting diodes (LEDs). In addition, 3D printed helmet and armor are demonstrated to have both flame-retardant and mechanical protective properties, which make them more desirable for future applications.

## 2. Results and Discussion

The well-defined BM architecture and excellent mechanical properties of the natural nacre inspired us to design novel multifunctional composites [7]. However, replication of the hierarchical BM structure in complex shapes is challenging because the accurate alignment of high loading ceramic fillers is difficult to control in 3D printing processes. Previous studies on the nacre-inspired structures by 3D printing emphasized on the single mechanical property by using pure polymers, multimaterials, or composites with low loading of fillers [14, 19, 20].

**Figure 1:**
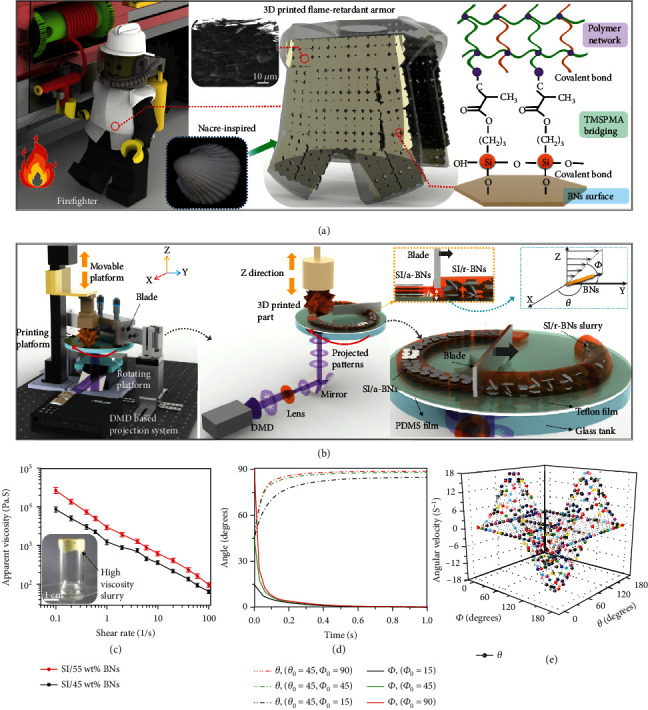
(a) Schematic diagram shows the 3D printed fire-retardant armor on a LEGO firefighter with nacre-inspired aligned BNs along with a SEM image of the BM structure. The bonding between TMSPMA grafted BNs with polymer network is also presented. (b) Setup of the rotation blade casting-assisted 3D printing technology, the alignment of BN generated by blade casting in the projection-based stereolithography process, and the alignment mechanisms. (c) Viscosity with the shear rate for different loadings of BNs, the inset image shows the SI/55 wt% BN slurry in a glass bottle. (d) Changes to *θ*and ∅ with respect to time for different BNs with different initial values of ∅_0_and *θ*_0_. (e) Angular velocity $\dot{\theta }$ with the angles of BNs with respect to the casting direction.

Here, a rotation-blade casting-assisted 3D printing method based on stereolithography (SL) using a digital micromirror device (DMD) was developed to build nacre-inspired structures. The 3D printed structures with a-BNs in the photocurable polymer (SI) show significantly improved mechanical and thermal properties for integrated mechanical and thermal protection applications (Figure 1(a)). Crack deflection at the large area of platelet interfaces contributes to the toughening of the natural nacre structure [21]. In this work, BNs were used due to their large surface area (Figure S1). BNs were further surface modified with 3-(trimethoxysilyl)propyl methacrylate (TMSPMA) to graft on the platelet surface (Figure S2). TMSPMA will react with the photocurable monomer during the light projection, which will promote homogeneous distribution (Figure S6) and strengthen the interface bonding to improve the load transfer during the bridging and pull-outs of BN on the crack (Figure 1(a)). Besides, the increase of surface roughness after TMSPMA grafting will enhance the shear strength during BNs' pull-outs (Figure S4).

Figure 1(b) shows the *rbc*-3D printing setup to build nacre-inspired structures (see Materials and Methods). A doctor blade (height 20 mm) and a rotation platform (gap 100 *μ*m) were used to enable the alignment of BNs in the highly viscous slurry (see methods) (Figures 1(b) and 1(c) and S5). Jeffery's equations were used to study the angular velocity and angle changes with time for BNs under the rotation blade casting $\dot{\emptyset }=-\left(\dot{\gamma }/r_{e}^{2}+1\right)\left(r_{e}^{2}\sin^{2}\emptyset +\cos^{2}\emptyset \right)$, $\dot{\theta }=\left(r_{e}^{2}-1/r_{e}^{2}+1\right)\left(\dot{\gamma }/4\right)\sin2\emptyset \sin2\theta \text{s}$, and (gap *h* = 100 *μm*, shear rate: 72.5 s^−1^), $\dot{\gamma \;}$is the shear rate, *r*_*e*_is BNs' aspect ratio, and ∅ and *θ* are the angles of the platelets with reference to the casting direction (see methods), respectively. The results show that it takes a similar time for BNs with different initial angles ∅_0_ to orientate to the casting direction, and all of this will be achieved within 1 s (Figure 1(d)). The angular velocity $\dot{\emptyset }$ has the maximum and minimum value for the platelets that are perpendicular (∅ = 90°) and parallel (∅ = 0°) to the casting direction, respectively (Figure S5). It takes a similar time for the BNs with the same initial angle *θ*_0_ = 45° and different ∅_0_ to align to the shear plane (*θ* = 90°) (Figure 1(d)). The angular velocity $\dot{\theta }$ reaches the maximum value when ∅ = 45° and *θ* = 45° (Figure 1(e)) [22]. The period of platelet rotation is given by $T=\left(2\pi /\dot{\gamma }\right)\left(r_{e}^{2}+1/r_{e}\right)$, demonstrating that the relaxation time is oppositely proportional to the shear rate. Thus, we can achieve a large shear rate to effectively align the BNs with a fast casting speed and a small gap. The efficiency of the BN alignment with the gap between the blade and the substrate and the casting speed was discussed in supporting information (Figure S5).

### 2.1. 3D Printing of Nacre and the Study of Toughing Mechanisms

**Figure 2:**
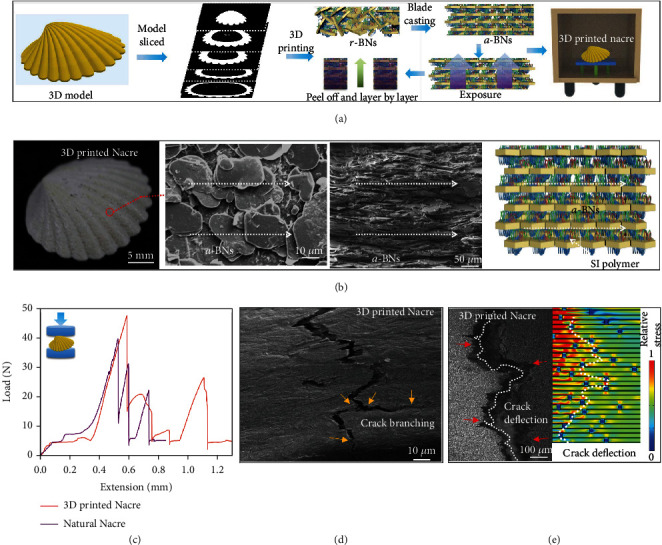
(a) Projection image patterns were generated by slicing the nacre model for 3D printing, and the BNs were aligned by the shear force during the rotation blade-casting; the selective light exposure (purple part) will cure the composites. (b) SEM images of 3D printed nacre demonstrating the accurate control of the alignment of BNs. (c) Comparison of the load-displacement curves of natural nacre and 3D printed nacre. (d) Crack branching in 3D printed nacre after fracture. (e) Crack deflection in the SEM image and the simulation by using COMSOL Multiphysics.

The 3D printing process is shown in Figure 2(a) and explained in detail in the experimental section. Figure 2(b) shows the SEM images of the BM structure of the 3D-printed nacre. The load-displacement diagram of the 3D-printed nacre with a-BNs under compression shows similar behavior to the natural nacre (Figure 2(c)) [15]. It is well known that there are multiple length scales of crack deflection and branching to suppress crack growth in natural nacre [23]. We found similar crack branching behavior and crack deflection in the 3D printed nacre with laminated structures (Figures 2(d) and 2(e)). The experimental results of crack deflection in the SEM images consistent well with the simulation in COMSOL Multiphysics (Figure 2(e)). Besides the primary energy dissipating by crack branching and deflection, we found that the a-BNs bridging and pull-outs also contribute to the excellent mechanical properties. A detailed structural analysis to understand the toughing mechanisms of 3D printed a-BNs is discussed in the following section.

**Figure 3:**
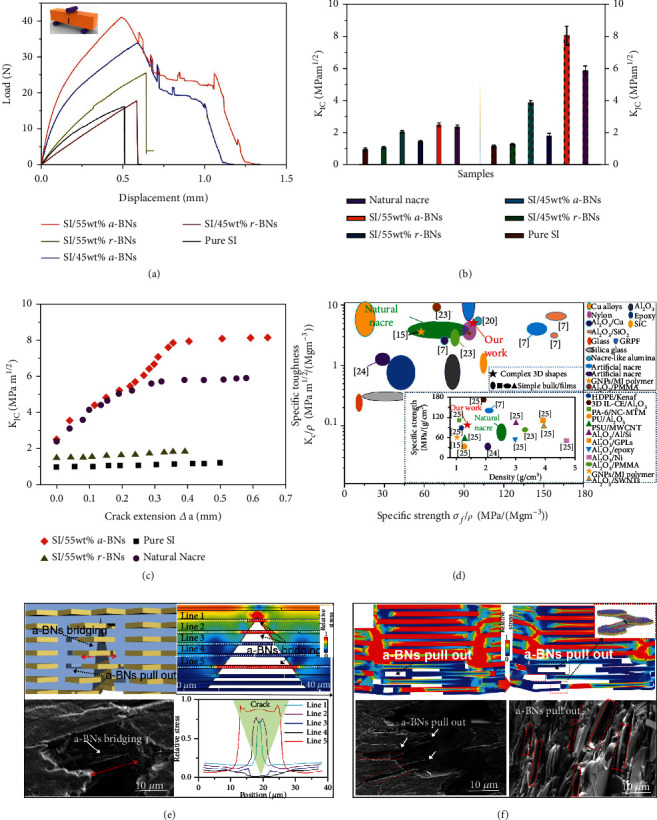
(a) Three-point bending tests for 3D printed structures with pure SI, SI/r-BNs, and SI/a-BNs. (b) Fracture toughness *K*_*IC*_ (resistance to crack initiation) and *K*_*JC*_ (resistance to crack propagation) of the 3D-printed nacre with different orientations and loadings of BNs and the natural nacre. (c) *K*_*JC*_ vs. crack extension of the 3D printed nacre-inspired structure and natural nacre. (d) Specific toughness and strength of the nacre-inspired structures with complex shapes and simple bulk/films (inset shows the lightweight and strong properties of our 3D printed nacre-inspired structure) [7, 21, 27, 29, 30]; SEM images and schematic diagram show the (e) a-BN bridging and (f) a-BN pull-outs in the crack during the crack deflection and stress distribution on the crack simulated in COMSOL Multiphysics.

To study the reinforcement mechanisms of the nacre-inspired structure, first, the fracture toughness is analyzed, which determines the reliability of the structure to resist fracture [7]. The measurements show that the 3D-printed structures with pure SI and random BNs (r-BNs) exhibit linear elastic responses with a catastrophic failure (Figure 3(a)). In comparison, several peaks for a-BNs were observed, demonstrating the crack growth resistance and the pull-outs of BNs (discussed in Figure 3(f)). Because of its high aspect ratio, the strengthening efficiency of a-BNs is strongly affected by their arrangement and the interconnect bonding [24]. The maximum load during the bending test for a-BNs without surface modification is 59.7% lower than that for a-BNs grafted with TMSPMA, demonstrating the enhancement of interfacial strength and load transfer by covalent bonding (Figure S8). The fracture toughness (*K*_*IC*_) for the 3D printed structures with 55 wt% a-BNs (~2.53 MPa m^1/2^) is larger than that of natural nacre (~2.4 MPa m^1/2^). With the crack growth, the fracture toughness (*K*_*JC*_) will change with the crack extension (△*α*) [25, 26]. In natural nacre, a rising crack resistance curve (*R*-curve) was generated due to the increase of *K*_*JC*_ with the crack extension [21, 27, 28]. The 3D-printed SI/a-BN structure exhibits similar *R*-curve behavior, while no *R*-curves were observed for the 3D-printed structures with pure SI and SI/r-BNs due to easy crack propagation [7]. It demonstrates that a-BNs will absorb energy during the crack growth and inhibit the critical cracking [29] (Figure 3(c)). *K*_*JC*_ of the nacre-inspired a-BNs reaches ~8.14 MPa m^1/2^ at the end of crack deflection (*E* = 2.37 *GPa*), which is much larger than *K*_*JC*_ of the natural nacre (~5.9 MPam^1/2^) (Figure 3(b)) [7]. In comparison, *K*_*JC*_ for the r-GN composite (~1.81 MPa m^1/2^) shows little improvement. Our 3D-printed nacre exhibits larger specific toughness and specific strength than those of natural nacre (*ρ*_2_ = 2.58 *g*/*cm*^3^) due to the decreased density (*ρ*_1_ = 1.49 *g*/*cm*^3^) (Figure 3(d)) [7]. The 3D-printed nacre-inspired structure with a-BNs develops its toughening capability primarily during crack growth.

To study the reinforcement mechanism, SEM images after the 3-point bending test show that the crack tip will be deflected when it reaches the a-BNs during crack formation. During the crack growth, the a-BNs were observed to act as bridges to span the crack (Figure 3(e)) [27]. Simulation by COMSOL Multiphysics shows that the load carried by the a-BN bridging in the crack is 4-10 times higher than the BNs and polymer matrix out of the crack (Figure 3(e)). This load carried by the bridging a-BNs and the energy absorption will inhibit the crack propagation. The simulation by COMSOL Multiphysics is consistent with SEM images, showing that the a-BNs are pulled out from the polymeric matrix [31] (Figure 3(f)). Previous reports indicate that the fracture via breakage of platelets shows more brittle behavior than the fracture via pull-out of platelets [5]. The pull-outs of a-BNs demonstrate that the average aspect ratio (*s* ~36-50) is below *s*_*c*_, which is much larger than *s*_*c*−*nacre*_ (9-12.5), allowing for a considerable increase of interface area [5]. The bonding of TMSPMA grafted a-BNs with polymer matrix provides interfacial hardening during BN tile sliding, pull-outs, and crack formation in the 3-point bending test. After the pull-outs, the load carried by a-BNs will dramatically decrease, but new a-BN bridging will form. The a-BN bridging and pull-outs will carry the load and absorb energy and trigger crack deflection, enhancing the fracture toughness (Figures 3(e) and 3(f)). The nacre-inspired alignment of BNs by *rbc*-3D printing promotes a-B bridging and pull-outs and results in the *R*-curve behavior for excellent mechanical properties [32] (Figure S9).

### 2.2. Study of Thermal Management Capability of the 3D Printed Nacre-Inspired Structure

**Figure 4:**
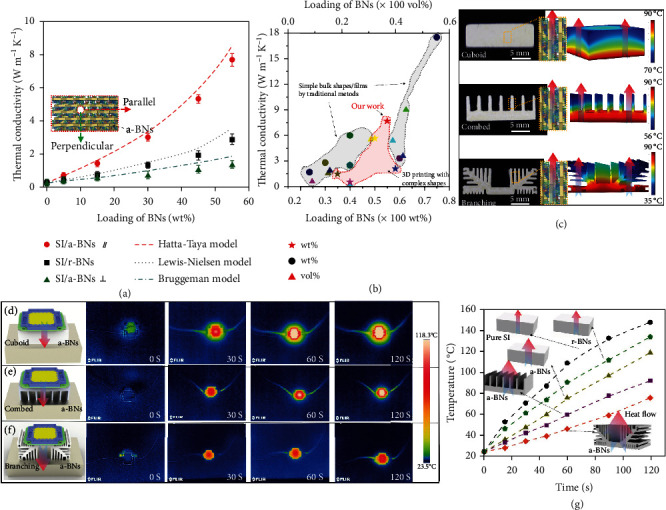
(a) Comparison of theoretical and experimental thermal conductivity for the samples with random BNs, perpendicular and parallel to the alignment for the aligned BNs. (b) Thermal conductivity of nacre-inspired composites with a-BNs by 3D printing and traditional methods (the symbols demonstrate different types of loading (wt% and vol%) of BNs). (c) 3D printed heat sink with structural optimization for high power LEDs; IR images for the heat control of LEDs connected to the 3D printed heat sink with cuboid (d), combed (e) and branching (f) shapes. (g) Comparison of temperature changes with time during the heat dissipation process for 3D printed heat sink with different shapes and orientation of BNs.

Effective thermal control has attracted much attention for a wide range of applications such as LEDs, integrated electronic devices (computer chips), and energy-related devices [33–35]. For example, LED cooling requires an optimized design with high thermal conductivity and easy processability [36]. Previous studies show that the alignment of fillers by various methods (vacuum filtration, electric/magnetic field, hot-pressing, etc.) will result in improved thermal conductivity [37–39], but the structures are limited to thin films or simple bulk shapes. We present here optimized thermal control of complex shapes with vertically aligned BNs and accordingly improved thermal conductivity by the *rbc*-3D printing method. Enhanced thermal conductivity with an increasing concentration of BNs was observed for all of the composites (Figure 4(a)). The thermal conductivity of 3D printed structures with 55 wt% a-BNs shows a maximum value of 7.69 W.m^−1^ K^−1^, which is improved by 25.5 times than pure SI (0.29 W.m^−1^ K^−1^). The structure also shows a highly anisotropic heat conduction performance, with ~5.8 times in the inplane direction compared with the out-of-plane direction. The high thermal conductivity is attributed to the low BN-BN interface thermal resistance by the interconnected a-BNs in the casting direction (Figure 2(b)) [33]. Such a pathway will facilitate the construction of the heat-transfer network and dramatically increase thermal conductivity [40]. However, in the direction perpendicular to the alignment in r-BN composites, there is low thermal conductivity due to the polymeric interlayers and high interface thermal resistance between matrix and BNs.

To study the mechanisms of enhanced thermal conductivity for 3D-printed structures, several models were used and analyzed. The Hatta-Taya model matches well with the thermal conductivity parallel to the alignment for 3D printed a-BN composites. The Hatta-Taya model assumes that the BNs are completely aligned [41]: *K*_*C*_ = *K*_*m*_ + [*V*_*f*_ (*K*_*f*_ − *K*_*m*_)*K*_*m*_]/[(*K*_*f*_ − *K*_*m*_)(1 − *V*_*f*_)*S*_*i*_ + *K*_*m*_], where *S*_*i*_ = (*πT*)/(4*D*), *D* (diameter), *T* (thickness), and *V*_*f*_ (volume fraction) are related to BNs. *K*_*c*_, *K*_*f*_, and *K*_*m*_ are the thermal conductivity of SI/a-BNs, BNs, and SI, respectively. The SEM images reveal that the a-BNs were highly interconnected and well-stacked in the 3D printed structure (Figure 2(b)). A large contact area between a-BNs will prominently reduce the thermal contact resistance. The agreement between experimental and theoretical results demonstrates good alignment of BNs by the *rbc*-3D printing process. The experimental result of thermal conductivity for SI/r-BNs agrees well with the Lewis-Nielsen model [41, 42]: *K*_*c*_/*K*_*m*_ = (1 + *ABV*_*f*_)(1 − *B*Ψ*V*_*f*_), *B* = (*K*_*f*_/*K*_*m*_ − 1)(*K*_*f*_/*K*_*m*_ + *A*), where *A* is the generalized Einstein constant, *A* = 30; *B* is related to the thermal conductivity of BNs (*K*_*f*_) and SI matrix (*K*_*m*_); Ψ = 1 + ((1 − *φ*_*m*_)/*φ*^2^_*m*_)*f*, and the maximum packing fraction is *φ*_m_ = 0.52 for r-BNs. The apparent thermal conductivity of K ~ 16 Wm^−1^ K^−1^ has been extracted for r-BNs [42]. For the heat transport in SI/r-BN structure, the disconnection between adjacent platelets will lead to a low thermal conductivity value. The out-of-plane thermal conductivity of the SI/a-BNs matches well with the Bruggeman model [43]: 1 − *V*_*f*_ = [(*K*_*c*_ − *K*_*f*_)/(*K*_*m*_ − *K*_*f*_)](*K*_*m*_/*K*_*c*_)^1/3^. The Bruggeman equation considers the dilute suspension of platelets in the composites. The thermal conductivity of SI/r-BNs is higher than the out-of-plane thermal conductivity for SI/a-BNs, attributed to the block of heat transport by the polymer matrix in between r-BNs. Thus, by controlling the alignment of BNs in 3D printing, various thermal conductivities can be achieved.

Heat accumulation from high-power electronics may result in equipment damage and reduces the lifetime of the equipment. To address this problem, optimized heat-sink shapes with vertical a-BNs were fabricated using *rbc*-3D printing. After evaluation, our 3D printed structures with a-BNs exhibit the highest thermal conductivity compared with other 3D printing methods (Figure 4(b) (the triangular shape is vol%, and the star and circular shapes are wt%) and Table S2 in supporting information)). The structures with higher thermal conductivity by traditional methods have a limitation of being constrained to simple bulk shapes. 3D printed heat sinks with different shapes (cuboid, combed, and branching) for a 10 W LED chip were fabricated to compare their thermal management performance. An infrared (IR) camera was used to monitor the changes in the surface temperature of the lighted LED chip with time (Figures 4(c)–4(f)). A comparison shows that the surface temperature of the LED for simple bulk shape with SI/a-BNs is 15.1°C and 29.0°C lower than those of SI/r-BNs and pure SI, respectively (Figure 4(g)). Further, structural optimization shows that the LED chip with the 3D printed branching shape sink with a-BNs displays a much lower temperature compared with the ones in cuboid and comb shapes. The surface temperature is only 75.1°C with the branching shape, which is 16.3°C and 43.2°C lower than that of comb and simple cuboid shapes, respectively, and 72.2°C lower than the pure SI with cuboid shape. These results demonstrate that the shape optimized structures with 3D-printed a-BNs can guarantee effective heat dissipation to increase the lifetime of LEDs.

### 2.3. Study of the Flame-Retardant Property of the 3D Printed Nacre-Inspired Structure

Previous studies on flame-retardant structures cannot provide mechanical protection and are limited to be films of simple bulk shapes (Table S1). The flame-retardant test on the natural nacre shows a crack after 5 s burning (Figure S13). In contrast, the 3D-printed structures with nacre-inspired a-BNs show better performance (sustaining 24 s and 50 s burning for a 3D-printed helmet and armor, respectively). First, the heat resistant property was studied by the thermogravimetric analysis (TGA) (Figure S14). The results show that the “heat resistance index *T*_HRI_” for SI/55 wt% BNs is 264.8°C, which is much higher than that of pure SI (148.2°C) (*T*_HRI_ = 0.49 × [*T*_5_ + 0.6 × (*T*_30_ − *T*_5_)]),where *T*_5_ and *T*_30_ are the temperature of 5 wt% and 30 wt% mass loss [44]).

**Figure 5:**
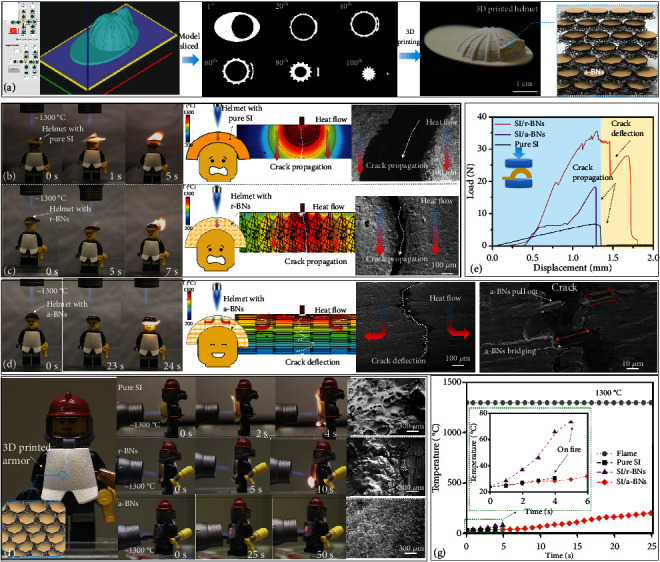
(a) 3D printing of a protective helmet with nacre-inspired a-BNs for firefighter. (b)–(d) Flame-retardant tests, demonstration of heat flow, and SEM images of cracks for the 3D printed helmet with pure SI, r-BNs, and a-BNs. (e) Load-displacement curves of the 3D printed helmet under compression. (f) Flame-retardant test for 3D printed armor and SEM images after burning for armors with pure SI, r-BNs, and a-BNs, respectively. (g) Temperature change from a thermocouple in between the armor and the LEGO firefighter body for the back of the 3D printed armor with pure SI, SI/r-BNs, and SI/a-BNs.

To study mechanical protection and flame-retardant properties, a helmet and armor for a LEGO firefighter were fabricated by *rbc*-3D printing to custom-fit the body shape (Figure 5(a)). The helmet (“thickness” 1 mm) fabricated by pure SI caught fire immediately (1 s) when it contacts the 1300°C flame, while the helmet with a-BNs shows the best performance (24 s before catching fire) compared with r-BNs (5 s) and pure SI (1 s). The a-BNs will redirect heat flow due to the anisotropic thermal conductivity, compared with the direct penetration of heat flow for pure SI and SI/r-BN structures (Figures 5(b) and 5(c)). In addition, the helmet with a-BNs shows a higher load at fracture compared with r-BNs and pure SI (Figure 5(e)). The improved strength is attributed to crack deflection, a-BNs bridging, and pull-outs (Figure 5(d)) compared with the crack propagation in helmets with r-BNs and pure SI (Figures 5(b) and 5(c)). Thus, the 3D printed helmet with a-BNs provides the LEGO firefighter with integrated mechanical and thermal protection. The flame-retardant properties of the 3D-printed armors with pure SI, r-BNs, and a-BNs were also tested. The 3D-printed armor with a-BNs (“thickness” 4 mm) did not catch fire with a longer time exposure (50 s), which demonstrates its flame retardant capability, while the armors with the pure SI and r-BNs catch fire at 3 s and 5 s, respectively (Figure 5(f)). A thermocouple was placed between the 3D-printed armors and the LEGO firefighter body. The temperature on the back of the armor with a-BNs increases to 193°C after 25 s, which is 1107°C lower than the flame temperature, demonstrating the heat-shield and thermal protection capability (Figure 5(g)). The SEM images of the 3D printed armor with the pure SI after burning show the formation of a porous structure with the expansion of the thickness during the burning of the layer-fabricated polymer. A porous structure and cracks were also observed on the surface of the r-BN armor after burning. In comparison, the a-BNs remained closely packed, and no expansion/porous was observed after the combustibility test (Figures 5(f) and S16). The hundreds of dense layers of a-BNs with a high melting temperature (~2973°C) are among the main reasons for its outstanding flame-retardant property [2, 45]. The anisotropic thermal conductivity also contributes to the heat-shielding through the redirection of heat flow in the plane direction (Figure 5(d)). During exposure to flame, the protective layers of a-BNs will prevent heat flow and oxygen from entering the inner layers [2, 46]. The a-BNs will also act as a physical barrier to hinder the foaming and shape-changing of the nacre-inspired structures.

To summarize, a rotation blade casting-assisted 3D printing process was developed to build nacre-inspired alignment of high-loading BNs into arbitrary complex structures. The fabricated 3D structures exhibit outstanding mechanical, thermal control, and flame-retardant properties. The structure with a-BNs shows higher fracture toughness and specific strength than natural nacre. Fundamental mechanism study shows that the excellent mechanical property is attributed to the crack deflection, a-BNs bridging, and the large shear force during BN pull-outs enhanced by the covalent bonding between TMSPMA and polymer matrix. The structure shows 5.8 times anisotropic thermal conductivity and 25.5 times enhancement compared with pure SI. The high heat dissipation performance and the capability of 3D printing provide us with an efficient way to address the heat accumulation problem in electronic devices. The size of the 3D-printed structures fabricated by the rbc-3D printing technology will be affected by the constructed 3D printer's size. In addition to rotation, the blade casting technology base on a linear motion can also be used in constructing large-scale 3D printers. The 3D printed helmet and armor with nacre-inspired a-BNs show both mechanical and thermal protection, demonstrating potential applications in military, fire-fighting equipment, and mechanical and aerospace engineering.

## 3. Materials and Methods

### 3.1. Preparation of SI Photocurable Resin and Boron Nitride Composites

BN powder (Dandong Rijin Science and Technology Co., Ltd., *purity* > 99.5%, sizes ~18-25 *μ*m, thickness~ 500 nm) was dried in the oven at 150°C for 24 hours. Before mixing BNs with the photocurable solution, the dried platelets were functionalized with 3-(trimethoxysilyl)propyl methacrylate (TMSPMA) using a grafting strategy [47]. First, “mixture 1” was made by dissolving TMSPM (4 mL) in ethanol (200 mL). The acetic acid solution was made by a mixture of acetic acid (4 mL) with DI water (36 mL). Then, mixture 1 was mixed with the as-prepared acetic acid solution to prepare the TMSPMA solution. The BN powders (~2.4 g) were mixed with the TMSPMA solution and put in the ultrasonic bath for 24 h. Large amounts of ethanol and water were used to clean the platelets, which were then dried in an oven. Photocurable resin (SI500, SI) was purchased from EnvisionTEC (Dearborn, MI). To prepare the SI/BNs slurry, appropriate weight ratios of BNs and SI were used to study the effect of loading of fillers on multifunctional properties (Figure 3(b)). Wet ball milling for 1 hour (200 Rpm) was used to homogeneously disperse BNs in the SI polymer resin. After ball milling, the slurry was put in a vacuum for 1 hour to evacuate the gas bubbles. An FT/IR 420 Fourier transform infrared spectrometer (JASCO, Easton, MD) was used to collect the Fourier-transform infrared spectroscopy (FTIR) spectra (Figure S3). The scanning electron microscopy (SEM) images were taken using a JSM-7001F microscope.

### 3.2. Investigation of the Rotation of BNs under Blade Casting

The coordinate system of BNs with the shear force direction is shown in Figure 1(b). Jeffery's equations for the angular velocity of platelet in simple shear flow are [22],$\;\dot{\emptyset }=-\left(\dot{\gamma }/r_{e}^{2}+1\right)\left(r_{e}^{2}\sin^{2}\emptyset +\cos^{2}\emptyset \right)$,$\dot{\theta }=\left(r_{e}^{2}-1/r_{e}^{2}+1\right)\left(\dot{\gamma }/4\right)\sin2\emptyset \sin2\theta$, (gap *h* =100 *μ*m, shear rate: 72.5 s^−1^), $\dot{\gamma }$is the shear rate, *r*_*e*_is the BNs' aspect ratio, ∅ and *θ* are the angles of the platelets with reference to the casting direction, and *θ* = 90° when the platelet lies in the shear plane. Integrated previous equations with time, $\tan\emptyset =\left(1/r_{e}\right)\tan\left[-\dot{\gamma }t\left(r_{e}/r_{e}^{2}+1\right)+\tan^{-1}\left(r_{e}\tan\emptyset_{0}\right)\right]$, tan*θ* = Cr_e_/(*r*_*e*_^2^sin^2^∅+cos^2^∅)^1/2^, where ∅_0_ is the initial angle, *C* is the constant that is related with the initial values of ∅_0_ and *θ*_0_, and *t* is the time.

### 3.3. 3D Printing of Nacre, Flame-Retardant Helmet, and Armor

The models of nacre, armor, and helmet were created in SolidWorks and sliced to different patterns for mask image projection (Figure 2(a)) [48, 49]. The photocurable SI/BN slurry was deposited on a Teflon film attached to the glass substrate (Figure 1(a)). Then, the rotation of the platform generated a shear force between the blade and the substrate, leading to the alignment of BNs. The slurry after casting was selectively cured with projection DMD patterns (light intensity: 3.16 mW cm^−2^, resolution of the DMD chip: 1024 × 768), and the aligned BNs were fixed to form the nacre-inspired structure in each layer (Figure 2(a)). After curing by projection light, strong covalent chemical bonds (CH_2_–CH_2_ group) connect the TMSPMA-grafted BNs with the SI matrix. The thickness of each layer was controlled to be 50 *μ*m. After curing, a new layer of the solidified pattern attached to the previous layer; then, the stage moved up to peel it off from the Teflon film. The stage then moved down after blade-casting to fabricate additional layers (Figure 2(b)). A total of 100 layers took ~35 mins to fabricate the artificial nacre.

A total of 80 and 120 layers were used to print the armor and helmet, respectively (Figure 5(a)). The mechanical and thermal properties of the 3D-printed armor/helmet were tested and analyzed after fabrication. A Digital 2 Channels K-Type Thermometer was placed between the armor and the LEGO firefighter body, which measured the temperature of the back of the armor during the combustion tests. To reduce experimental errors, five samples were fabricated and tested for each case.

### 3.4. 3D Printing of Heat Exchange Structure for Thermal Control

Different models of heat exchange structures were designed in SolidWorks and sliced into different mask images with the DMD-based stereolithography software. Then, 3D printing of heat exchange structures was performed through a layer-by-layer fabrication process. Vertically aligned BNs were prepared by horizontally aligning BNs by the blade casting. The 3D-printed structure was then rotate by 90 degrees and attached to the LED surface.

### 3.5. Mechanical Testing

The mechanical properties of natural nacre were collected from reference 7. The mechanical properties were tested by Instron 5492 Dual Column Testing Systems (Instron, MA, USA). A compressive velocity of 1 mm min^−1^ and a maximum displacement of 5 mm were set in the static compression test. The samples with notches were 3D-printed, and compressive velocity of 1 mm min^−1^, a maximum displacement of 4 mm was used for the single-edge notched bend (SENB) tests. *K*_*IC*_ was analyzed according to equation [7, 50, 51]:

(1) $$\begin{array}{c} K_{\text{IC}}=\frac{P∙S}{B∙W^{3/2}}∙f\left(\frac{a}{W}\right),f\left(\frac{a}{W}\right)=\frac{3∙{\left(a/W\right)}^{1/2}∙\left[1.99-\left(a/W\right)∙\left(1-\left(a/W\right)\right)∙215-3.93∙\left(a/W\right)+2.7∙{\left(a/W\right)}^{2}\right]}{2∙\left(1+2∙\left(a/W\right)\right)∙{\left(1-\left(a/W\right)\right)}^{3/2}}, \end{array}$$

where the maximum load (*P*), the thickness of the specimen *W* = 1 *mm*, the width *B* = 2.05 *mm*, the support span *S* = 4 *mm*, and the notch depth *a* = 0.3 *mm*.

*K*_*JC*_ was analyzed from two different portions (the elastic and plastic) from the *J*-integral calculation [7]. *J* = *J*_el_ + *J*_pl_, where *J*_el_ = *K*_IC_^2^/*E*′ is the elastic portion from the linear elastic fracture mechanics. *J*_pl_ = 2*A*_pl_/*B*(*W* − *a*) is the plastic portion, in which *A*_*pl*_ is the area of plastic portion in the load-displacement curve. Thus, *K*_*JC*_ is related to *J* values: *K*_JC_ = (JE′)^1/2^, where *E*′ = *E*(1 − *v*)^2^ that is related to Young's modulus (*E*) and the Poisson's ratio (*v*). *E*′can be replaced by *E* due to the limited influence on *K*_*JC*_.

The crack extension ∆*α* is related to the equation [7, 52]: *α*_*n*_ = *α*_*n*−1_ + (*W* − *α*_*n*−1_/2)(*C*_*n*−_*C*_*n*−1_/*C*_*n*_), *C*_*n*_ = *u*_*n*_/*f*_*n*_, and ∆*α* = *α*_*n*_ − *α*, where *C*_*n*,_*α*_*n*_, *f*_*n*_, and *u*_*n*_ are the complaisance, crack length, force, and displacement at each point after the departure of the crack, respectively. Flexural strength is determined by *σ* = 3FL/2bd^2^ (13), for the 3D-printed sample with 55 wt%, *F* = 30.8 *N*, support span length *L* = 10 *mm*, width *b* = 3.23 *mm*, thickness *d* = 1 *mm*, the flexural strength is 143 MPa, and the specific strength is 95.9 MPam^1/2^/(Mgm^−3^), which is also slightly larger than the specific strength of natural C.plicata nacre (~60-90 MPam^1/2^/(Mgm^−3^)) (Figure S10).

### 3.6. Thermal Conductivity and Flame-Retardancy Tests

The thermal diffusivity *D* was tested with a laser flash principle using a Netzsch LFA 457 system. The thermal conductivity: *κ* = *ρ*∙*D*∙*C*_*p*_, where the heat capacity *C*_*p*_ and density *ρ* were from the test [53, 54]. A mini Jet Pencil Gun Torch with 1300°C flame was used to test the fire-retardant property of the 3D printed structures with pure SI, SI/r-BNs, and SI/a-BNs. The fire retardancy testing process was recorded on a Canon 6D camera. In each test, the blue flame was kept the same, and the distance between the torch and 3D printed armor was kept the same (20 mm); so, the flame contacts the sample's surface. The flame direction is perpendicular to the surface of each sample.

### 3.7. Simulation of Mechanical/Thermal Properties

The models of composites with different orientations of BNs were designed in SolidWorks and imported into COMSOL Multiphysics. A compressive force (200 N) was applied to study the crack deflection, load carried by a-BNs bridging and pull-outs. Stresses applied in both cases were 200 N/6*e* − 3 *m*^2^ = 33.3 *kPa*. The moduli of SI and BNs were set to be 500 MPa and 100 GPa, respectively. The interface between the BN elements and the background epoxy was the contact boundary with contribution from friction. The static Coulomb friction model was used, and the Tangential force method was penalty. For the simulation of heat flow, the thermal conductivity of SI and BNs was set to be 0.29 W.m^−1^ K^−1^ and 200 W.m^−1^ K^−1^, respectively.

## Data Availability

All data needed to evaluate the conclusions in the paper are present in the paper and/or the supporting information.

## Supplementary Materials

Supplementary Materials Table S1: comparison of mechanical protection property and shape complexity for boron nitride nanoplatelets (BNs) and nacre-inspired flame-retardant structures. Figure S1: SEM images of aligned BNs and magnified view of BNs. Figure S2: (a) representation of the surface modification procedure of BNs by 3-(trimethoxysilyl)propyl methacrylate (TMSPMA). (b) Schematic diagram shows the alignment of BNs in photocurable monomer and the covalent bonding between TMSPMA and photocurable monomer. Figure S3: FTIR spectrum of pure BNs and surface modified BNs by 3-(trimethoxysilyl)propyl methacrylate (TMSPMA). The diagram on the right shows the corresponding chemical bonding on the FTIR spectrum. Figure S4: SEM images of the original BNs (unmodified) and the TMSPMA-grafted BNs. Comparison of stress distribution during the sliding of adjacent BNs for the unmodified BNs and the TMSPMA-grafted BNs simulated by COMSOL Multiphysics. Figure S5: study of the efficiency of alignment of BNs with the gap between the doctor blade and the substrate, (a) 100 *μ*m, (b) 300 *μ*m,and (c) 500 *μ*m. Figure S6: SEM images of SI/rBNs, SI/a-BNs with the unmodified BNs, and SI/a-BNs with the TMSPMA-grafted BNs. Figure S7: changes of cure depth with the fraction of BNs. Figure S8: comparison of 3-point-bending tests for 3D printed a-BNs with the unmodified BNs and the TMSPMA-grafted BNs. Figure S9: crack deflection, a-BN bridging, and pulling out for 3D printed nacre-inspired structures with TMSPMA grafted a-BNs. Figure S10: the standard three-point-bending tests were performed to study the flexural strength of the 3D-printed structures. Figure S11: compression test of the 3D printed nacre with aligned BNs. Table S2: comparison of thermal conductivity of our work with other 3D printing and traditional methods. Figure S12: setup for the test of thermal control structures with 3D printed shapes. Figure S13: flame-retardant test of natural nacre. Figure S14: TGA tests of pure SI, BNs, and SI/55 wt% BNs and the comparison of their heat resistance index. Figure S15: testing of the surface temperature of a LEGO body under flame with the protection of a 3D printed armor by a thermometer. Figure S16: SEM images of the pure SI, SI/r-BNs, and SI/a-BNs after the combustion test, the blue part demonstrates the shape of 3D printed armor, and the red part is under the flame. Figure S17: low magnification of SEM image shows the well aligned BN in the 3D printed structure.
