# Supplementary material for: 3D Printing of Nacre-Inspired Structures with Exceptional Mechanical and Flame-Retardant Properties
Source: Research (Wash D C). 2022 Jan 27;2022:9840574. doi: 10.34133/2022/9840574 (PMC8817185; doi:10.34133/2022/9840574)
Supplement: Supplementary Materials — Table S1: comparison of mechanical protection property and shape complexity for boron nitride nanoplatelets (BNs) and nacre-inspired flame-retardant structures. Figure S1: SEM images of aligned BNs and magnified view of BNs. Figure S2: (a) representation of the surface modification procedure of BNs by 3-(trimethoxysilyl)propyl methacrylate (TMSPMA). (b) Schematic diagram shows the alignment of BNs in photocurable monomer and the covalent bonding between TMSPMA and photocurable monomer. Figure S3: FTIR spectrum of pure BNs and surface modified BNs by 3-(trimethoxysilyl)propyl methacrylate (TMSPMA). The diagram on the right shows the corresponding chemical bonding on the FTIR spectrum. Figure S4: SEM images of the original BNs (unmodified) and the TMSPMA-grafted BNs. Comparison of stress distribution during the sliding of adjacent BNs for the unmodified BNs and the TMSPMA-grafted BNs simulated by COMSOL Multiphysics. Figure S5: study of the efficiency of alignment of BNs with the gap between the doctor blade and the substrate, (a) 100 μm, (b) 300 μm,and (c) 500 μm. Figure S6: SEM images of SI/rBNs, SI/a-BNs with the unmodified BNs, and SI/a-BNs with the TMSPMA-grafted BNs. Figure S7: changes of cure depth with the fraction of BNs. Figure S8: comparison of 3-point-bending tests for 3D printed a-BNs with the unmodified BNs and the TMSPMA-grafted BNs. Figure S9: crack deflection, a-BN bridging, and pulling out for 3D printed nacre-inspired structures with TMSPMA grafted a-BNs. Figure S10: the standard three-point-bending tests were performed to study the flexural strength of the 3D-printed structures. Figure S11: compression test of the 3D printed nacre with aligned BNs. Table S2: comparison of thermal conductivity of our work with other 3D printing and traditional methods. Figure S12: setup for the test of thermal control structures with 3D printed shapes. Figure S13: flame-retardant test of natural nacre. Figure S14: TGA tests of pure SI, BNs, and SI/55 wt% BNs and the [file 9840574.f1.zip › Revised-Supplemental Material.docx]

**Supplemental Material**

**3D Printing of Nacre inspired Structures with Exceptional Mechanical and Flame-retardant Properties**

Yang Yang1,*, Ziyu Wang2,*, Qingqing He1, Xiangjia Li3, Gengxi Lu4, Laiming Jiang4,5, Yushun Zeng4, Brandon Bethers1, Jie Jin5,6, Shuang Lin7, Siqi Xiao7, Yizhen Zhu3,8, Xianke Wu7,9, Wenwu Xu1, Qiming Wang10, Yong Chen 5,8,*.

1. Department of Mechanical Engineering, San Diego State University, 5500 Campanile Drive, San Diego, CA 92182, USA.
2. The Institute of Technological Sciences, Wuhan University, Wuhan 430072, China.
3. School for Engineering of Matter, Transport and Energy, Arizona State University, 551 E Tyler Mall, Tempe, AZ 85281, USA.
4. Department of Biomedical Engineering, University of Southern California,1042 Downey Way, Los Angeles, CA 90089, USA.
5. Epstein Department of Industrial and Systems Engineering, University of Southern California, 3715 McClintock Ave, Los Angeles, CA 90089, USA.
6. ShadeCraft Robotics Inc. Pasadena, CA, 91105, USA
7. Department of Chemical Engineering and Materials Science, University of Southern California, 925 Bloom Walk, Los Angeles, California 90089, USA
8. Department of Aerospace and Mechanical Engineering, University of Southern California, Los Angeles, CA 90089, USA.
9. School of Physics and Technology, Wuhan University, Wuhan 430072, China.
10. Sonny Astani Department of Civil and Environmental Engineering, University of Southern California, Los Angeles, CA 90089, USA.

Corresponding authors: [yyang10@sdsu.edu,](mailto:yyang10@sdsu.edu,) [zywang@whu.edu.cn;](mailto:zywang@whu.edu.cn;) yongchen@usc.edu.

**Table S1**. Comparison of mechanical protection property and shape complexity for boron nitride nanoplatelets (BNs) and nacre-inspired flame-retardant structures [1-14]


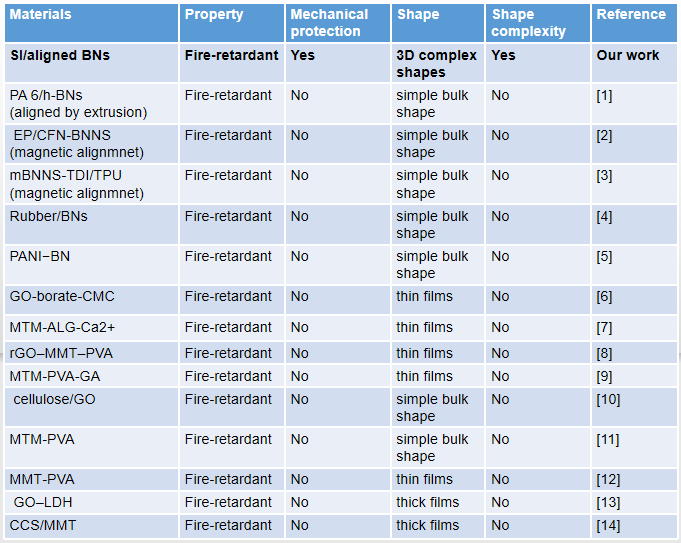


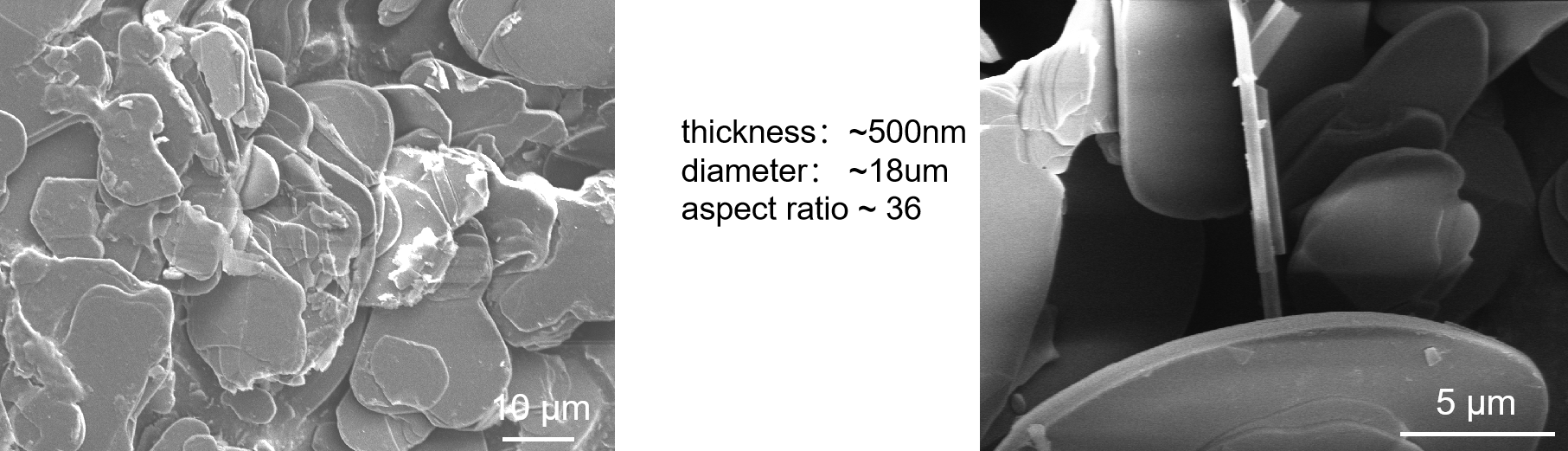


**Figure S1.** SEM images of aligned BNs and magnified view of BNs.

From Figure S1, the boron nitride nanoplatelets (BNs) were well-aligned. The thickness of each BN is 500 nm, and the diameter is around 18-25 μm with an aspect ratio of 36-50.


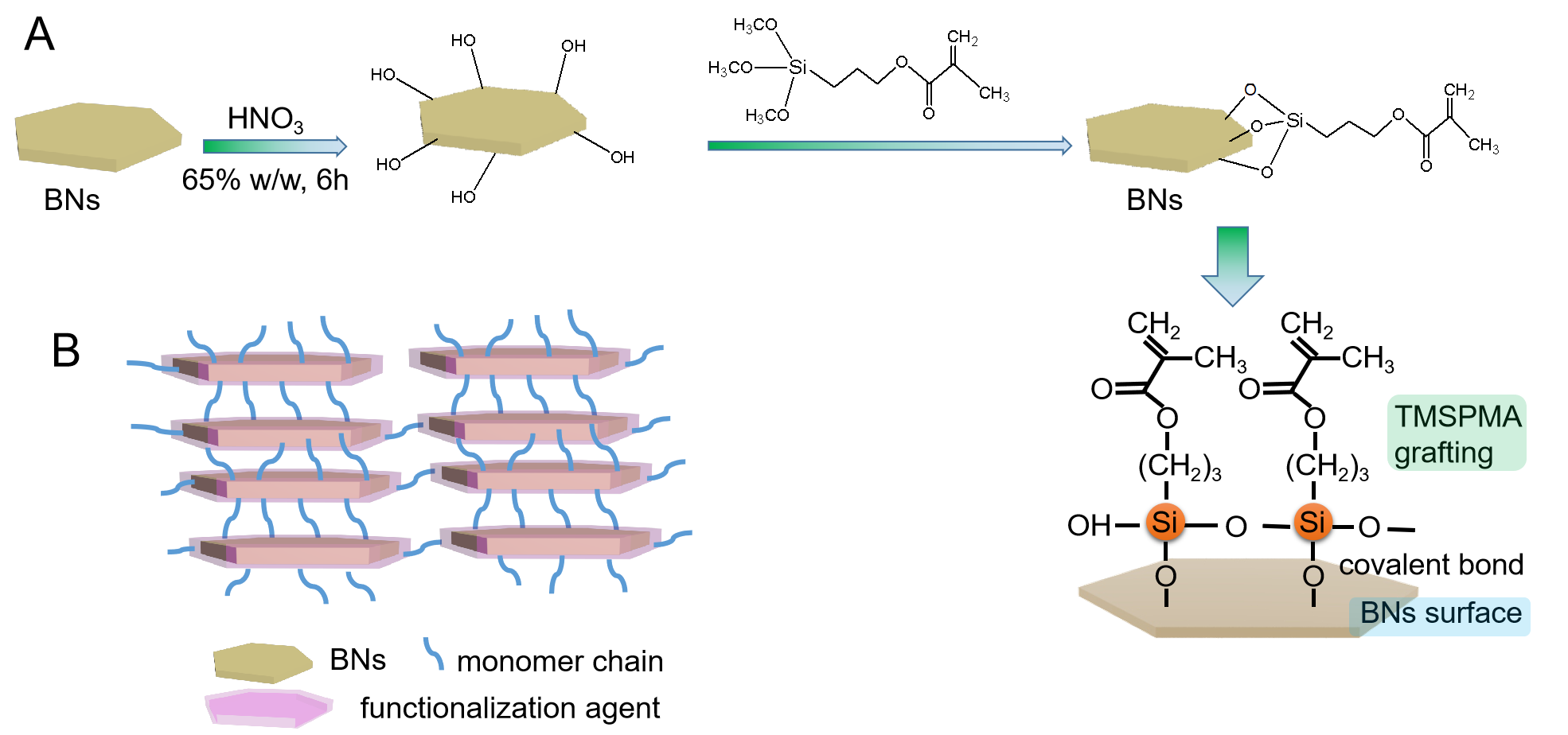


Figure S2. (A) Representation of the surface modification procedure of BNs by 3-(Trimethoxysilyl)propyl methacrylate (TMSPMA); and (B) schematic diagram shows the alignment of BNs in photocurable monomer and the covalent bonding between TMSPMA and photocurable monomer.


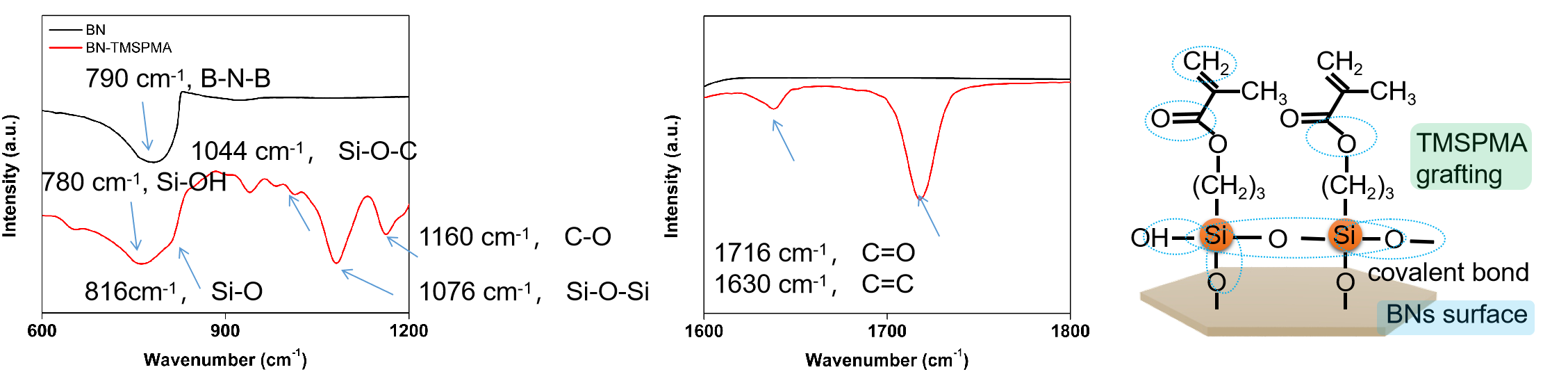


Figure S3. FTIR spectrum of pure BNs and surface modified BNs by 3-(Trimethoxysilyl)propyl methacrylate (TMSPMA). The diagram on the right shows the corresponding chemical bonding on the FTIR spectrum.

The interface bonding between the BNs and the photocurable resin (SI500, SI) polymer matrix is greatly important because it affects the load transfer and energy dissipation during the crack formation and deflection. The surface modification of BNs has been used to address low interfacial strength between interlayers of the artificial nacre. The covalent bonding shows higher performance than other types of bonding such as hydrogen bonding. The surface modification of BNs allows the use of chemical bonding to improve the interfacial strength between adjacent layers. The schematic diagram shows the surface modification process of BNs with the grafting of 3-(Trimethoxysilyl)propyl methacrylate (TMSPMA) through covalent bonding (Fig. S2A), which is discussed in the experimental section. The FTIR spectra of the reaction product s-BNs shows clear absorption at 780cm-1 (Si-OH), 816cm-1 (Si-O), 10446cm-1 (Si-O-C), 1076cm-1 (Si-O-Si), 1160cm-1 (C-O), 1630cm-1 (C=C) and 1716cm-1 (C=O)[15-17]. These absorption bands characterize the presence of TMSPMA on the surface of BNs (Supplementary materials, Fig. S2A). After the functionalization agent is covalently grafted to the BN platelet surface, the BNs are mixed with the photocurable monomer (SI polymer). During the 3d printing process, after curing by light, strong covalent chemical bonds (CH2–CH2 group) connect the modified BN with the photopolymer matrix. This matrix inclusion linkage enables effective stress transfer between BNs and SI polymer and facilitates the homogeneous distribution of BNs in the liquid resin.


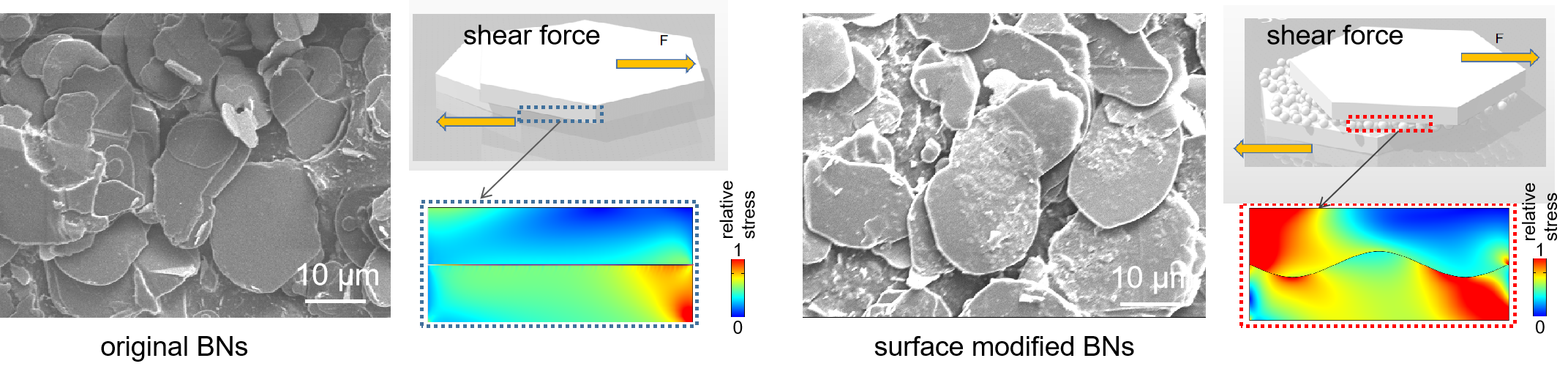


**Figure S4.** SEM images of the original BNs (unmodified) and the TMSPMA-grafted BNs. Comparison of stress distribution during the sliding of adjacent BNs for the unmodified BNs and the TMSPMA-grafted BNs simulated by Comsol Multiphysics.

Note that the surface roughness of the TMSPMA-grafted BNs is higher than that of the pure BNs (Figure S4). Both the unmodified BNs and the surface-modified BNs show the alignment parallel to the shear force direction. The increment of surface roughness will play an important role in enhancing shear force and energy during the pullout of a-BNs. From the simulation by Comsol Multiphysics, the high surface roughness of the TMSPMA-grafted BNs will lead to enhanced shear force during the sliding of adjacent BNs compared with the smooth surface of the unmodified BNs.


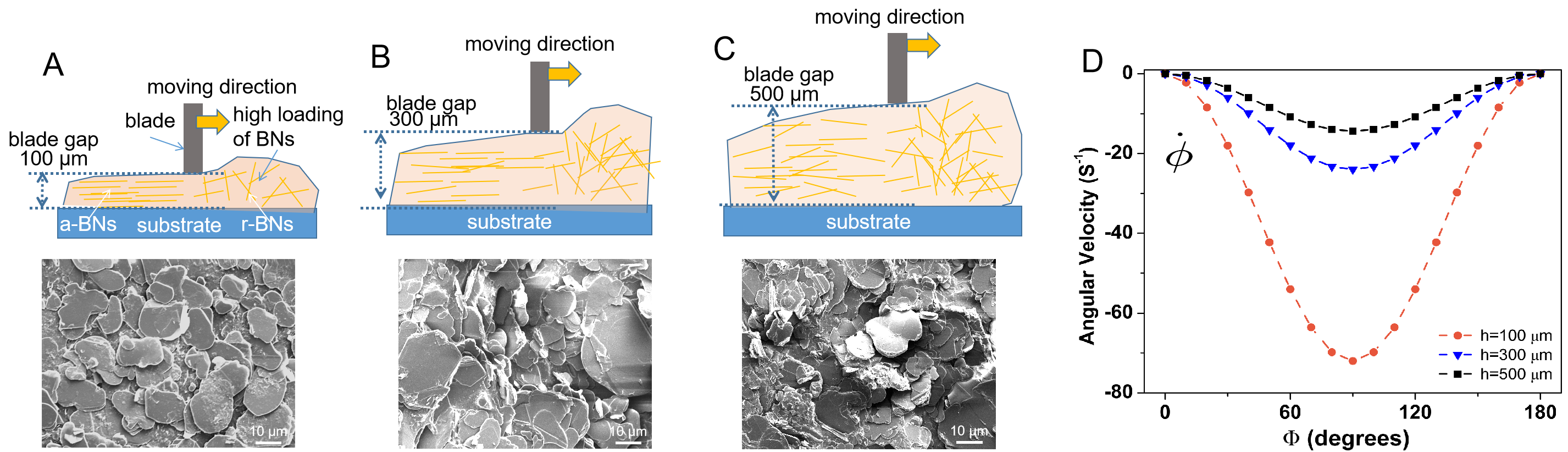


**Figure S5.** Study of the efficiency of alignment of BNs with the gap between the doctor blade and the substrate, (A) 100 μm; (B) 300μm; (C) 500 μm.

The alignment efficiency related to the gap between the blade and the substrate and the casting speed is shown in Figure S5. The alignment efficiency is attributed to the changes of shear rate with different gaps and velocities. , where is the shear rate, *v* is the velocity of the blade (7.25 mm/s), and *h* is the gap. From this equation, the shear rate is proportional to the gap with the same velocity. Jeffery’s equations for the angular velocity of a platelet in simple shear flow are . In the equation, is the shear rate and different, is the aspect ratio of the BN platelet, and are the angles of the platelet with respect to the flow direction, and when the platelet lies in the plane of shear. The angular velocity is higher for the smaller gap than the larger gap (Fig. S5D), which will generate the effective alignment of BNs at a shorter time. The SEM images show the results that agree well with the analysis. The alignment efficiency for a gap of 100 μm is higher than the gap of 500 μm due to the higher shear rate for a smaller gap.


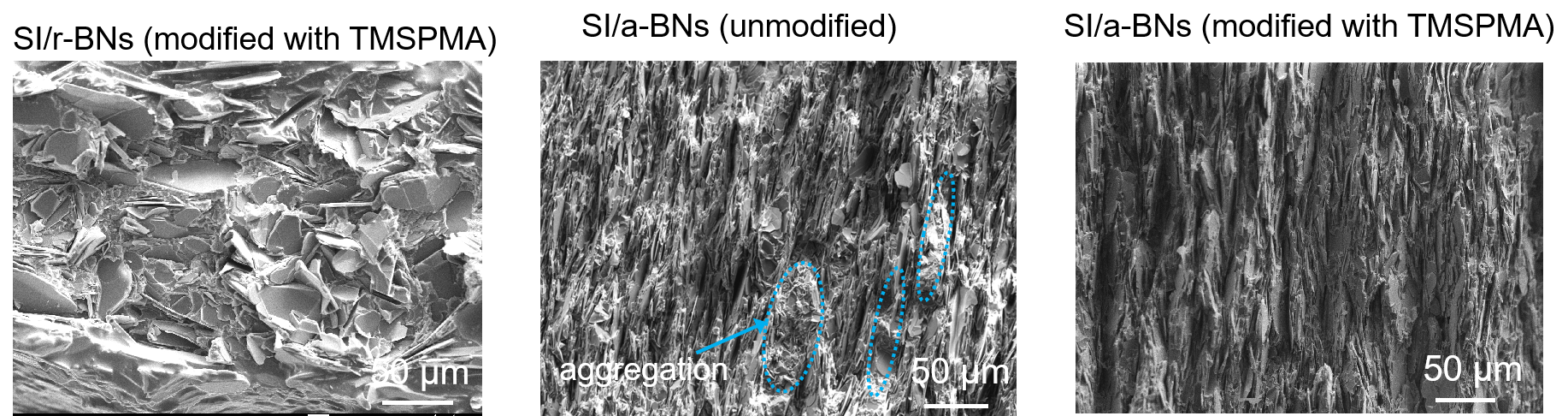


**Figure S6.** SEM images of SI/rBNs, SI/a-BNs with the unmodified BNs and SI/a-BNs with the TMSPMA-grafted BNs.

The BNs platelets show a well-flaked structure and smooth surface with an average lateral size of about 18 µm. The scanning electron microscope (SEM) images show that the surface modification improves the dispersity of BNs in the polymer resin (Fig. S6). There are aggregations of unmodified BNs in the polymer matrix, while the modified BNs were homogeneously dispersed in the polymer matrix (Figure S6). The aggregation of BNs will affect the alignment efficiency and lead to defects in the 3D-printed composites and the related poor mechanical property. After surface modification, the aggregation of the platelets was not observed, and the surface became rougher with small particles that appeared on the surface of BNs.


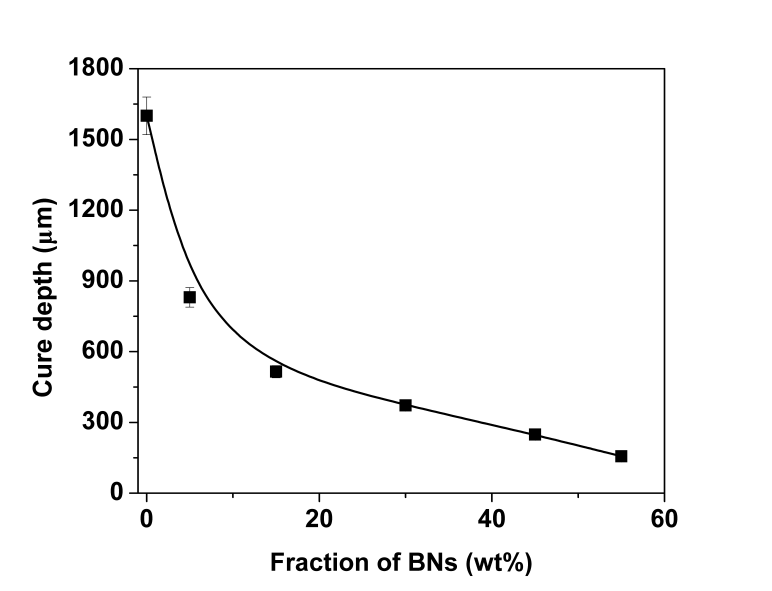


**Figure S7**. Changes of cure depth with the fraction of BNs

Figure S7 shows the decrease of the slurry’s cure depth with the increment of BNs loading in the SI/BNs slurry. The curing time was set as 30 s in all the tests. The resolution of the DMD chip (Texas Instrument, Dallas, TX) was 1024 × 768, and the output light intensity of the projection system was 3.16 mWcm-2. The results show that the cure depth dramatically decreases with the increment of the fraction of BNs.


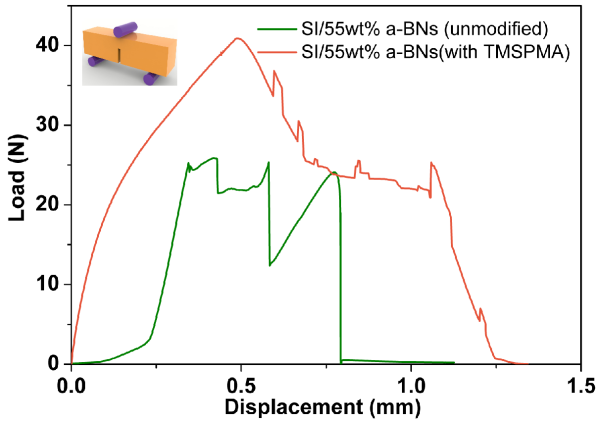


**Figure S8.** Comparison of 3-point-bending tests for 3D printed a-BNs with the unmodified BNs and the TMSPMA-grafted BNs.

The 3-point-bending test results for the 3D printed a-BNs structures with unmodified BNs and TMSPMA grafted BNs are shown in Fig. S8. The maximum load during the bending test for a-BNs without surface modification is 59.7% lower than that for a-BNs with TMSPMA, demonstrating the enhancement of well-distributed BNs and interfacial strength by covalent bonding between BNs, TMSPMA, and polymer matrix. The bonding between TMSPMA and polymer matrix leads to the improvement of interfacial shear strength, bonding energy, and load transfer between the BN platelets and the polymer matrix. Besides, the curve shows more peaks demonstrating crack deflection for the TMSPMA-grafted a-BNs than the unmodified a-BNs.


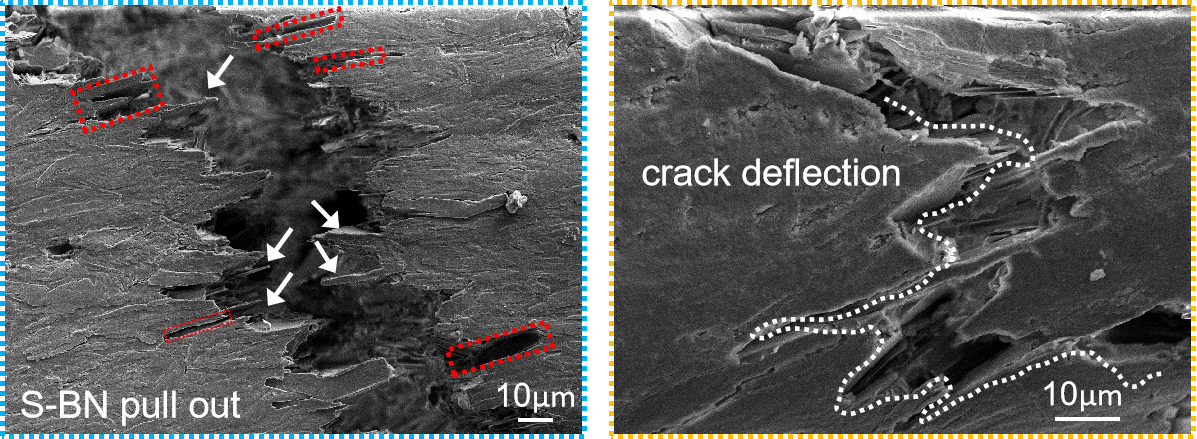


**Figure S9.** Crack deflection, a-BNs bridging, and pulling out for 3D printed nacre-inspired structures with TMSPMA grafted a-BNs.

Figure S9 shows obvious crack deflection, and BNs pulling out for the 3D-printed nacre-inspired structures with the TMSPMA-grafted a-BNs. The presence of BNs bridges ahead of the main growing crack leads to the formation of uncracked ligament bridging in the wake of the crack tip. Such bridges effectively span the crack and carry the load that would otherwise be used to promote macroscopic crack advance [18]. In addition, there is no fracture of the ceramic bricks; instead, there is some degree of ‘pull-out’ between the bricks that replicates the phenomena observed in natural nacre [19].


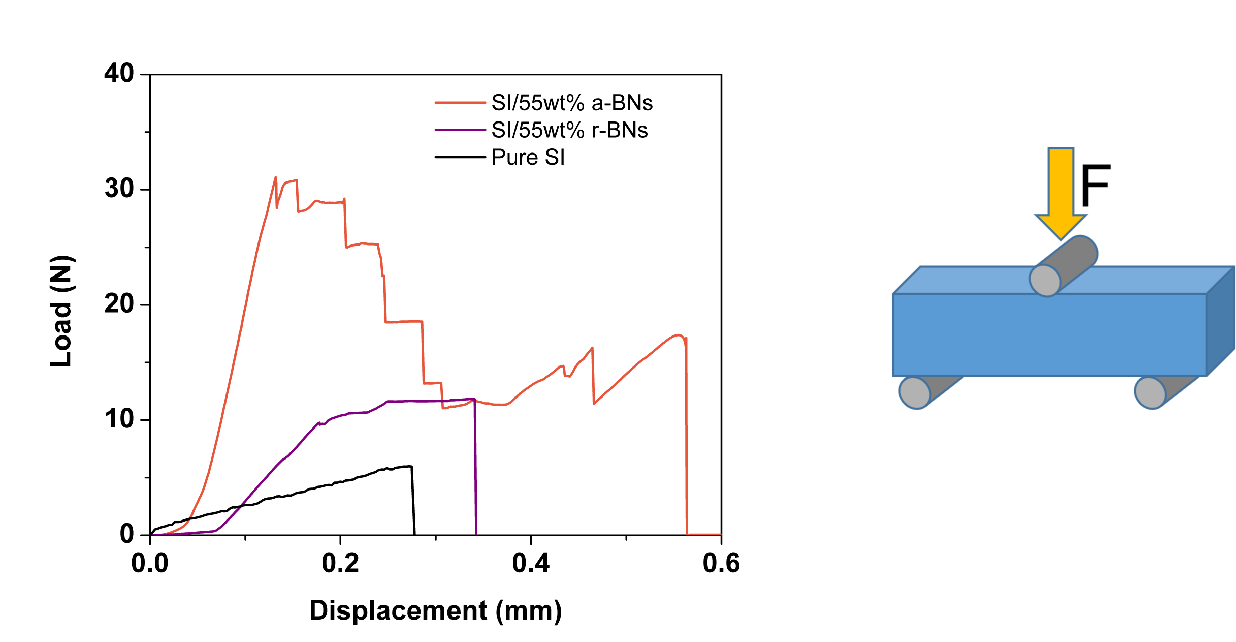


**Figure S10.** The standard three-point-bending tests were performed to study the flexural strength of the 3D-printed structures.

For a rectangular sample under a load in a standard three-point-bending setup (without a notch), the flexural strength will be: , in which *F* is the load at the fracture point, *L* is the length of the support span, *b* is the width, and *d* is the thickness of the sample. The test samples of SI/a-BNs were fabricated by the developed blade casting-assisted 3D printing process. The SI/r-BNs samples were built by modeling without blade casting. For the 3D printed samples with 55 wt%, *F*=30.8 N, *l*= 10 mm, *b*= 3.23 mm, and *d*= 1 mm, the flexural strength is 143 MPa, and the specific strength is 95.9 MPam1/2/(Mgm-3), which is slightly higher than that of natural C.plicata nacre (~60-90 MPam1/2/(Mgm-3)).


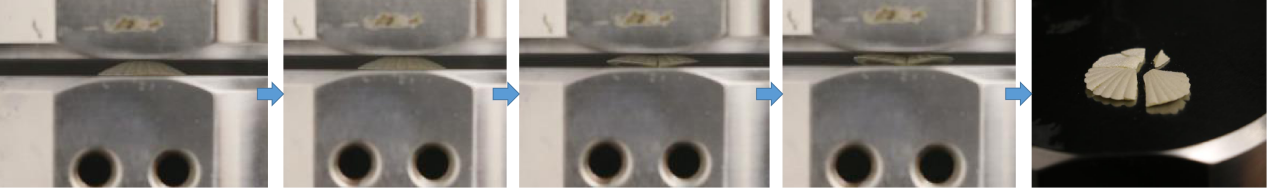


**Figure S11.** Compression test of the 3D printed nacre with aligned BNs.

Figure S11 shows the fracture of a 3D-printed nacre after compression. Note that the crack is focused on the tip and propagates after the compression. Under the external loading, the initial crack will be deflected by the a-BNs. Then BNs’ bridging will carry most of the load for the crack to act as a barrier for the crack propagation. The BNs’ bridging will dissipate a large amount of energy. When the loading further increases, the covalent bonding between BNs and TMSPMA as well as the polymer matrix stacking breaks. This will generate the pullout of the BNs in the crack, while the covalent bonding will absorb large amount of energy during the sliding and pulling out of BNs in the crack. The essence of the synergistic toughening effect is to simultaneously maximize the enhancement of the BNs’ bridging and strong covalent bonding. Consequently, the mechanical properties of SI/a-BNs nanocomposites are much improved.

**Table S2.** Comparison of thermal conductivity of Our work with other 3D printing and traditional methods [20-38].


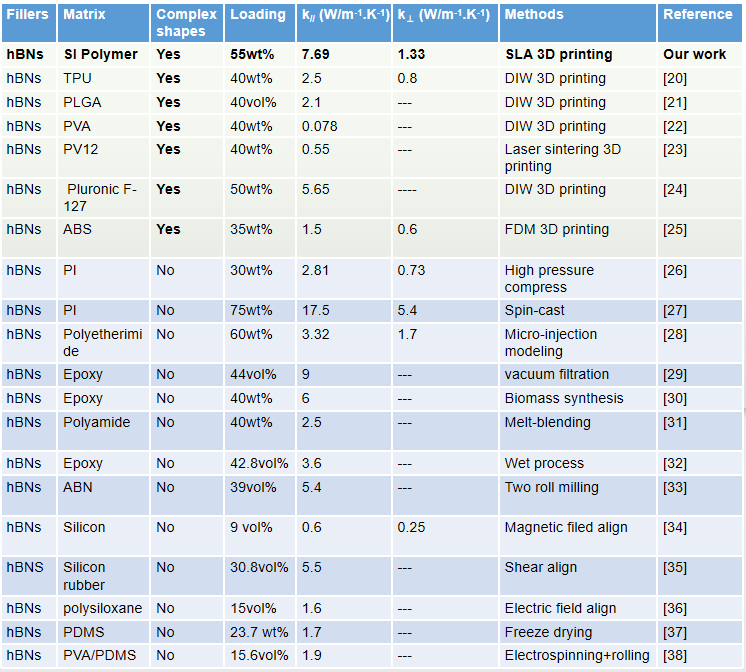


The performance and working reliability of electronic products are greatly affected by the thermal accumulation due to the miniaturization and multifunctionality integration of electronic devices. The fabrication of heat sink with complex shapes for optimized thermal control is challenging. We studied the anisotropic thermal conductivity in complex shapes by our developed rotate blade casting-assisted 3d printing. The 3D printing process provides tremendous opportunities for thermal-based topology optimization to be used in the optimal design of thermal components. The 3D-printed heat sink with different shapes (cuboid, combed, and branch) were fabricated, and the thermal control performance was measured.

The heat resistance index *THRI* values of the SI/55wt% BNs composites is 264.8 °C, far above that of pure SI (148.2 °C). The reason is that BN fillers will absorb heat, resulting in SI molecular chains degraded at a higher temperature.


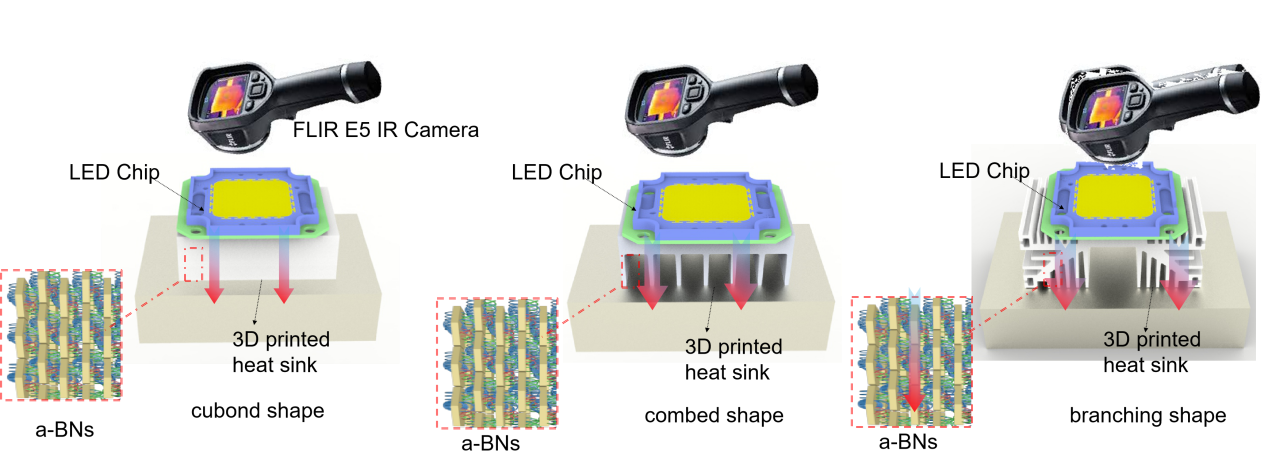


**Figure S12**. Setup for the test of thermal control structures with 3D printed shapes.


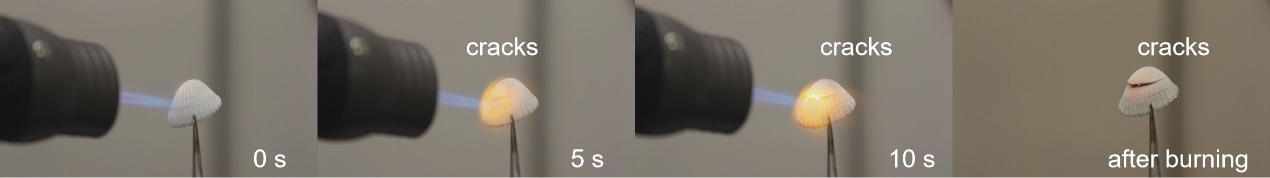


**Figure S13**. Flame-retardant test of natural nacre.


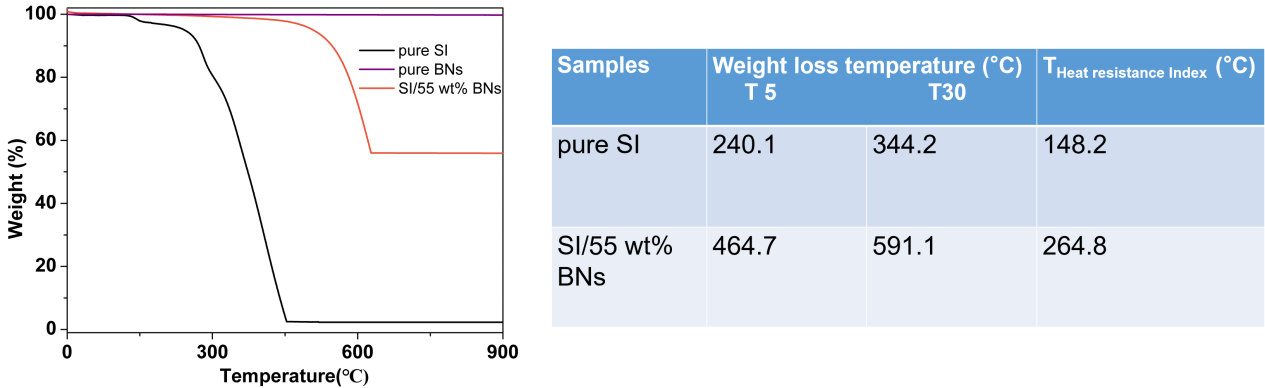


**Figure. S14**. TGA tests of pure SI, BNs and SI/55wt% BNs and the comparison of their heat resistance index.


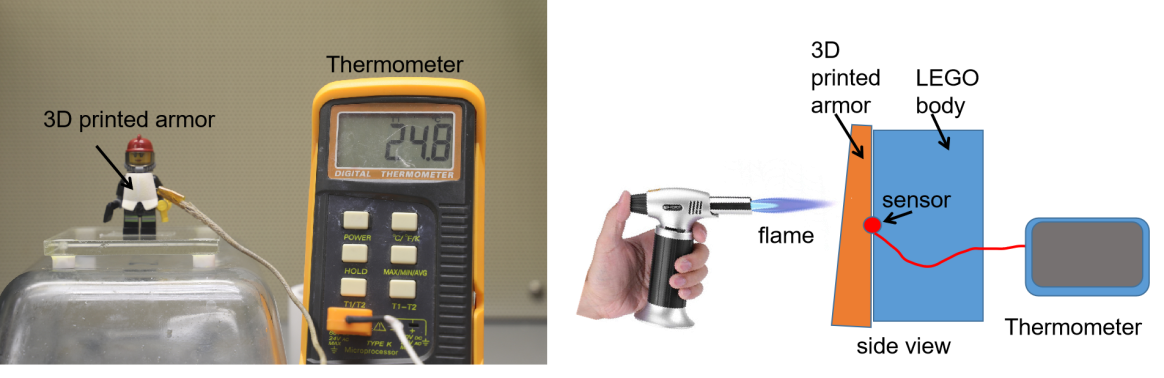


**Figure S15**. Testing of the surface temperature of a LEGO body under flame with the protection of a 3D printed armor by a thermometer.

A mini Jet Pencil Gun Torch with 1300℃ flame was used to test the fire-retardant property of the 3D printed structures with pure SI, SI/r-BNs, and SI/a-BNs. A camera (Canon 6D) was used to monitor the combustion behavior during the test. A thermometer (Digital 2 Channels K-Type) was used, and the sensor was put in between the back of 3D-printed armor and LEGO body to monitor the temperature changes (Fig. S13). In each test, the blue flame was kept the same, and the distance was kept so the flame contacted the surface of the sample. The flame direction was perpendicular to the surface of each sample.


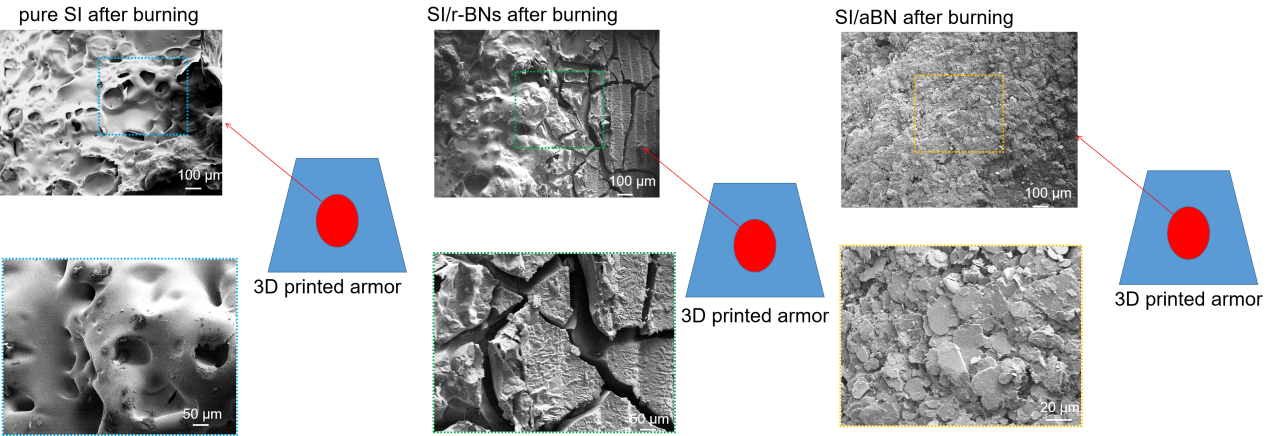


**Figure S16.** SEM images of the pure SI, SI/r-BNs, and SI/a-BNs after the combustion test, the blue part demonstrates the shape of 3D printed armor, and the red part is under the flame.

Different from conventional fire-retardant structures, the 3D-printed nacre-inspired structures show the capability in shape control for various complex surfaces. The Pure SI polymer can release gases that cause burning composite to foam, and then there are lots of porous structures emerged, which will promote oxygen access (Fig. S16 left). Thus, the 3D printed armor with pure SI cannot provide flame-retardant protection. When r-BNs were incorporated into SI, the flame-retardancy of the composite was efficiently enhanced; however, there are still porous structures and cracks formed on the surface (Fig. S16 middle). Furthermore, the SI/a-BNs composites show excellent flame-retardancy with the 55wt% BNs (Fig. S16 right), which resulted from the combined effect of flaky BNs and their alignment. On the one hand, the stability of BNs under high temperature and the alignment of the BNs with the anisotropic thermal conductivity will result in thermal protection; on the other hand, the formed a-BNs physical barrier can act on the surface of the matrix to hinder the foaming of burning composites. These two factors increase the flame-retardancy of SI/a-BNs composites [1].


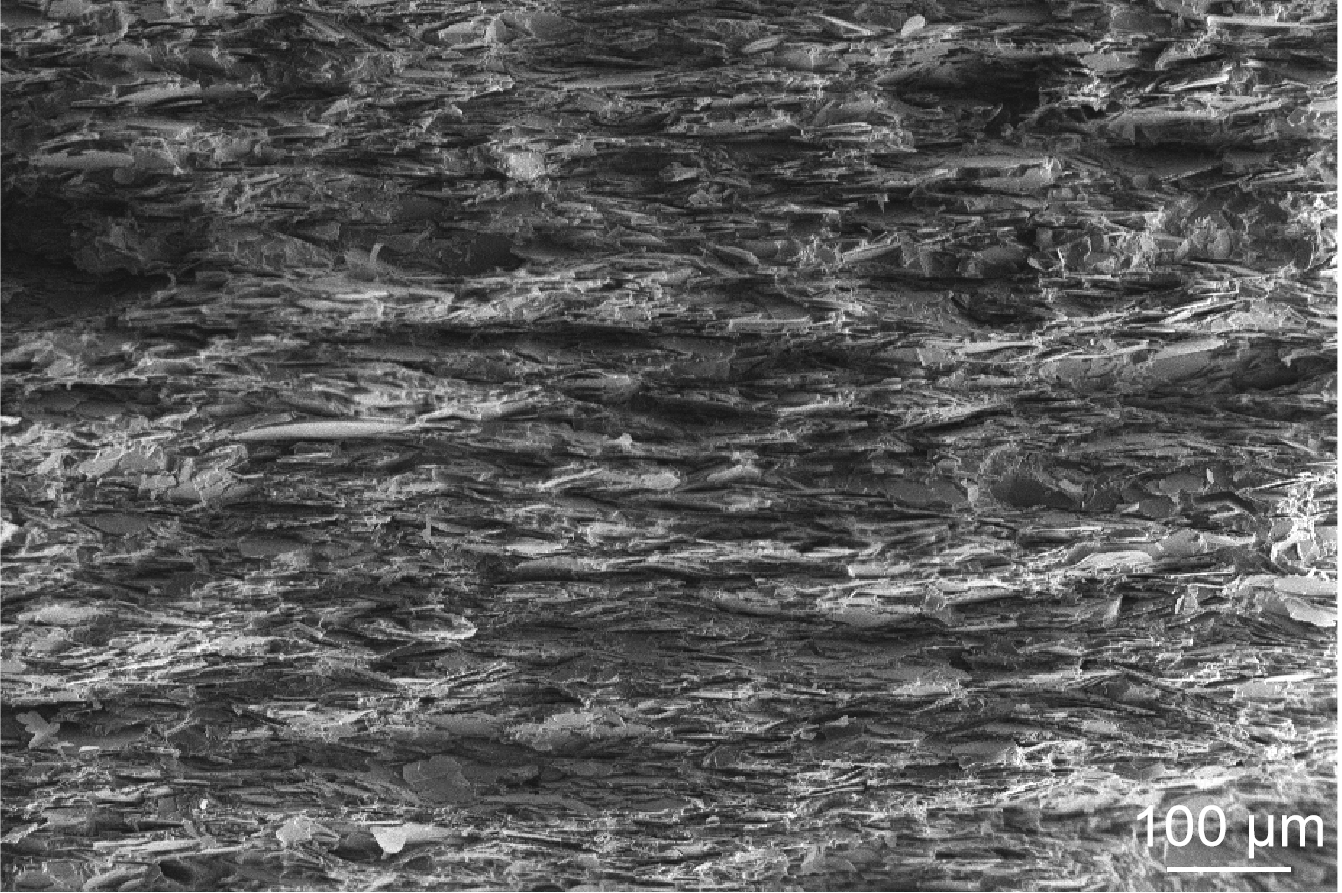


Figure S17. Low magnification of SEM image shows the well aligned BN in the 3D printed structure.

**References:**

1. Wang, L., L. Zhang, A. Fischer, Y. Zhong, D. Drummer, and W. Wu, *Enhanced thermal conductivity and flame retardancy of polyamide 6/flame retardant composites with hexagonal boron nitride.* Journal of Polymer Engineering, 2018. **38**(8): p. 767-774.

2. Zhang, Q., Z. Li, X. Li, L. Yu, Z. Zhang, and Z. Wu, *Preparation of cobalt ferrite nanoparticle-decorated boron nitride nanosheet flame retardant and its flame retardancy in epoxy resin.* Nano, 2019. **14**(05): p. 1950063.

3. Yuan, F., W. Jiao, F. Yang, W. Liu, Z. Xu, and R. Wang, *Surface modification and magnetic alignment of hexagonal boron nitride nanosheets for highly thermally conductive composites.* RSC advances, 2017. **7**(69): p. 43380-43389.

4. Wang, N., M. Zhou, J. Zhang, and Q. Fang, *Modified boron nitride as an efficient synergist to flame retardant natural rubber: preparation and properties.* Polymers for Advanced Technologies, 2020. **31**(9): p. 1887-1895.

5. Zhi, Y.-R., B. Yu, A.C.Y. Yuen, J. Liang, L.-Q. Wang, W. Yang, H.-D. Lu, and G.-H. Yeoh, *Surface manipulation of thermal-exfoliated hexagonal boron nitride with polyaniline for improving thermal stability and fire safety performance of polymeric materials.* ACS omega, 2018. **3**(11): p. 14942-14952.

6. Shahzadi, K., I. Mohsin, L. Wu, X. Ge, Y. Jiang, H. Li, and X. Mu, *Bio-based artificial nacre with excellent mechanical and barrier properties realized by a facile in situ reduction and cross-linking reaction.* ACS nano, 2017. **11**(1): p. 325-334.

7. Liang, B., H. Zhao, Q. Zhang, Y. Fan, Y. Yue, P. Yin, and L. Guo, *Ca2+ enhanced nacre-inspired montmorillonite–alginate film with superior mechanical, transparent, fire retardancy, and shape memory properties.* ACS Applied Materials & Interfaces, 2016. **8**(42): p. 28816-28823.

8. Ming, P., Z. Song, S. Gong, Y. Zhang, J. Duan, Q. Zhang, L. Jiang, and Q. Cheng, *Nacre-inspired integrated nanocomposites with fire retardant properties by graphene oxide and montmorillonite.* Journal of Materials Chemistry A, 2015. **3**(42): p. 21194-21200.

9. Walther, A., I. Bjurhager, J.-M. Malho, J. Pere, J. Ruokolainen, L.A. Berglund, and O. Ikkala, *Large-area, lightweight and thick biomimetic composites with superior material properties via fast, economic, and green pathways.* Nano letters, 2010. **10**(8): p. 2742-2748.

10. Xie, H., X. Lai, H. Li, J. Gao, X. Zeng, X. Huang, and X. Lin, *A highly efficient flame retardant nacre-inspired nanocoating with ultrasensitive fire-warning and self-healing capabilities.* Chemical Engineering Journal, 2019. **369**: p. 8-17.

11. Chen, S.-M., H.-L. Gao, X.-H. Sun, Z.-Y. Ma, T. Ma, J. Xia, Y.-B. Zhu, R. Zhao, H.-B. Yao, and H.-A. Wu, *Superior biomimetic nacreous bulk nanocomposites by a multiscale soft-rigid dual-network interfacial design strategy.* Matter, 2019. **1**(2): p. 412-427.

12. Ding, F., J. Liu, S. Zeng, Y. Xia, K.M. Wells, M.-P. Nieh, and L. Sun, *Biomimetic nanocoatings with exceptional mechanical, barrier, and flame-retardant properties from large-scale one-step coassembly.* Science advances, 2017. **3**(7): p. e1701212.

13. Yan, Y.X., H.B. Yao, L.B. Mao, A.M. Asiri, K.A. Alamry, H.M. Marwani, and S.H. Yu, *Micrometer‐Thick Graphene Oxide–Layered Double Hydroxide Nacre‐Inspired Coatings and Their Properties.* Small, 2016. **12**(6): p. 745-755.

14. Xie, H., X. Lai, Y. Wang, H. Li, and X. Zeng, *A green approach to fabricating nacre-inspired nanocoating for super-efficiently fire-safe polymers via one-step self-assembly.* Journal of hazardous materials, 2019. **365**: p. 125-136.

15. Li, Z., J. Shen, H. Ma, X. Lu, M. Shi, N. Li, and M. Ye, *Preparation and characterization of pH-and temperature-responsive hydrogels with surface-functionalized graphene oxide as the crosslinker.* Soft Matter, 2012. **8**(11): p. 3139-3145.

16. Simionescu, B., M. Olaru, M. Aflori, and C. Cotofana, *Silsesquioxane-based hybrid nanocomposite with self-assembling properties for porous limestones conservation.* High Performance Polymers, 2010. **22**(1): p. 42-55.

17. Cho, K., G. Wang, J. Fang, G. Rajan, M.H. Stenzel, P. Farrar, and B.G. Prusty, *Selective Atomic-Level Etching on Short S-Glass Fibres to Control Interfacial Properties for Restorative Dental Composites.* Scientific reports, 2019. **9**(1): p. 1-10.

18. Munch, E., M.E. Launey, D.H. Alsem, E. Saiz, A.P. Tomsia, and R.O. Ritchie, *Tough, bio-inspired hybrid materials.* Science, 2008. **322**(5907): p. 1516-1520.

19. Lin, A.Y.-M., P.-Y. Chen, and M.A. Meyers, *The growth of nacre in the abalone shell.* Acta Biomaterialia, 2008. **4**(1): p. 131-138.

20. Liu, J., W. Li, Y. Guo, H. Zhang, and Z. Zhang, *Improved thermal conductivity of thermoplastic polyurethane via aligned boron nitride platelets assisted by 3D printing.* Composites Part A: Applied Science and Manufacturing, 2019. **120**: p. 140-146.

21. Guiney, L.M., N.D. Mansukhani, A.E. Jakus, S.G. Wallace, R.N. Shah, and M.C. Hersam, *Three-dimensional printing of cytocompatible, thermally conductive hexagonal boron nitride nanocomposites.* Nano letters, 2018. **18**(6): p. 3488-3493.

22. Gao, T., Z. Yang, C. Chen, Y. Li, K. Fu, J. Dai, E.M. Hitz, H. Xie, B. Liu, and J. Song, *Three-dimensional printed thermal regulation textiles.* ACS nano, 2017. **11**(11): p. 11513-11520.

23. Yang, L., L. Wang, and Y. Chen, *Solid‐state shear milling method to prepare PA12/boron nitride thermal conductive composite powders and their selective laser sintering 3D‐printing.* Journal of Applied Polymer Science, 2020. **137**(23): p. 48766.

24. Liang, Z., Y. Pei, C. Chen, B. Jiang, Y. Yao, H. Xie, M. Jiao, G. Chen, T. Li, and B. Yang, *General, Vertical, Three-Dimensional Printing of Two-Dimensional Materials with Multiscale Alignment.* ACS nano, 2019. **13**(11): p. 12653-12661.

25. Quill, T.J., M.K. Smith, T. Zhou, M.G.S. Baioumy, J.P. Berenguer, B.A. Cola, K. Kalaitzidou, and T.L. Bougher, *Thermal and mechanical properties of 3D printed boron nitride–ABS composites.* Applied Composite Materials, 2018. **25**(5): p. 1205-1217.

26. Wang, H., D. Ding, Q. Liu, Y. Chen, and Q. Zhang, *Highly anisotropic thermally conductive polyimide composites via the alignment of boron nitride platelets.* Composites Part B: Engineering, 2019. **158**: p. 311-318.

27. Tanimoto, M., T. Yamagata, K. Miyata, and S. Ando, *Anisotropic thermal diffusivity of hexagonal boron nitride-filled polyimide films: effects of filler particle size, aggregation, orientation, and polymer chain rigidity.* ACS applied materials & interfaces, 2013. **5**(10): p. 4374-4382.

28. Lee, H.L., O.H. Kwon, S.M. Ha, B.G. Kim, Y.S. Kim, J.C. Won, J. Kim, J.H. Choi, and Y. Yoo, *Thermal conductivity improvement of surface-enhanced polyetherimide (PEI) composites using polyimide-coated h-BN particles.* Physical Chemistry Chemical Physics, 2014. **16**(37): p. 20041-20046.

29. Yu, C., J. Zhang, Z. Li, W. Tian, L. Wang, J. Luo, Q. Li, X. Fan, and Y. Yao, *Enhanced through-plane thermal conductivity of boron nitride/epoxy composites.* Composites Part A: Applied Science and Manufacturing, 2017. **98**: p. 25-31.

30. Wang, X.-B., Q. Weng, X. Wang, X. Li, J. Zhang, F. Liu, X.-F. Jiang, H. Guo, N. Xu, and D. Golberg, *Biomass-directed synthesis of 20 g high-quality boron nitride nanosheets for thermoconductive polymeric composites.* Acs Nano, 2014. **8**(9): p. 9081-9088.

31. Li, S., T. Yang, H. Zou, M. Liang, and Y. Chen, *Enhancement in thermal conductivity and mechanical properties via large-scale fabrication of boron nitride nanosheets.* High Performance Polymers, 2017. **29**(3): p. 315-327.

32. Kim, K., M. Kim, and J. Kim, *Thermal and mechanical properties of epoxy composites with a binary particle filler system consisting of aggregated and whisker type boron nitride particles.* Composites science and technology, 2014. **103**: p. 72-77.

33. Xue, Y., X. Li, H. Wang, F. Zhao, D. Zhang, and Y. Chen, *Improvement in thermal conductivity of through-plane aligned boron nitride/silicone rubber composites.* Materials & Design, 2019. **165**: p. 107580.

34. Yuan, C., B. Duan, L. Li, B. Xie, M. Huang, and X. Luo, *Thermal conductivity of polymer-based composites with magnetic aligned hexagonal boron nitride platelets.* ACS applied materials & interfaces, 2015. **7**(23): p. 13000-13006.

35. Kuang, Z., Y. Chen, Y. Lu, L. Liu, S. Hu, S. Wen, Y. Mao, and L. Zhang, *Fabrication of highly oriented hexagonal boron nitride nanosheet/elastomer nanocomposites with high thermal conductivity.* Small, 2015. **11**(14): p. 1655-1659.

36. Cho, H.-B., T. Nakayama, H. Suematsu, T. Suzuki, W. Jiang, K. Niihara, E. Song, N.S.A. Eom, S. Kim, and Y.-H. Choa, *Insulating polymer nanocomposites with high-thermal-conduction routes via linear densely packed boron nitride nanosheets.* Composites Science and Technology, 2016. **129**: p. 205-213.

37. Shen, H., C. Cai, J. Guo, Z. Qian, N. Zhao, and J. Xu, *Fabrication of oriented hBN scaffolds for thermal interface materials.* RSC advances, 2016. **6**(20): p. 16489-16494.

38. Chen, J., X. Huang, B. Sun, Y. Wang, Y. Zhu, and P. Jiang, *Vertically aligned and interconnected boron nitride nanosheets for advanced flexible nanocomposite thermal interface materials.* ACS applied materials & interfaces, 2017. **9**(36): p. 30909-30917.
