# Supplementary figures and images for: 3D Printing of Nacre-Inspired Structures with Exceptional Mechanical and Flame-Retardant Properties
Source: Research (Wash D C). 2022 Jan 27;2022:9840574. doi: 10.34133/2022/9840574 (PMC8817185; doi:10.34133/2022/9840574)

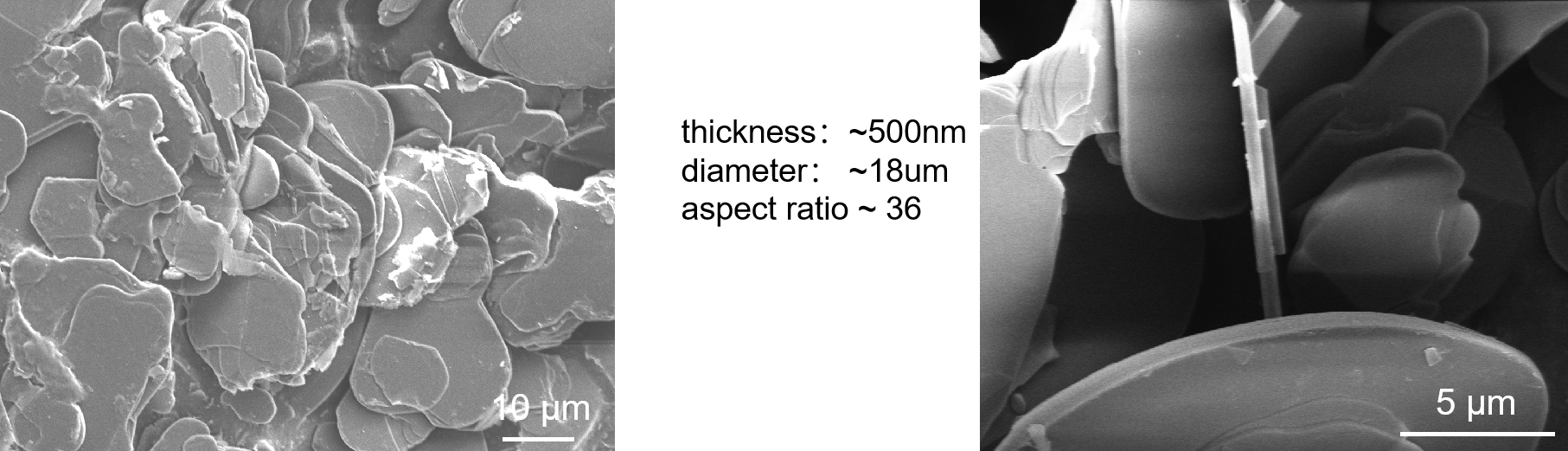

Supplement: Supplementary Materials — Table S1: comparison of mechanical protection property and shape complexity for boron nitride nanoplatelets (BNs) and nacre-inspired flame-retardant structures. Figure S1: SEM images of aligned BNs and magnified view of BNs. Figure S2: (a) representation of the surface modification procedure of BNs by 3-(trimethoxysilyl)propyl methacrylate (TMSPMA). (b) Schematic diagram shows the alignment of BNs in photocurable monomer and the covalent bonding between TMSPMA and photocurable monomer. Figure S3: FTIR spectrum of pure BNs and surface modified BNs by 3-(trimethoxysilyl)propyl methacrylate (TMSPMA). The diagram on the right shows the corresponding chemical bonding on the FTIR spectrum. Figure S4: SEM images of the original BNs (unmodified) and the TMSPMA-grafted BNs. Comparison of stress distribution during the sliding of adjacent BNs for the unmodified BNs and the TMSPMA-grafted BNs simulated by COMSOL Multiphysics. Figure S5: study of the efficiency of alignment of BNs with the gap between the doctor blade and the substrate, (a) 100 μm, (b) 300 μm,and (c) 500 μm. Figure S6: SEM images of SI/rBNs, SI/a-BNs with the unmodified BNs, and SI/a-BNs with the TMSPMA-grafted BNs. Figure S7: changes of cure depth with the fraction of BNs. Figure S8: comparison of 3-point-bending tests for 3D printed a-BNs with the unmodified BNs and the TMSPMA-grafted BNs. Figure S9: crack deflection, a-BN bridging, and pulling out for 3D printed nacre-inspired structures with TMSPMA grafted a-BNs. Figure S10: the standard three-point-bending tests were performed to study the flexural strength of the 3D-printed structures. Figure S11: compression test of the 3D printed nacre with aligned BNs. Table S2: comparison of thermal conductivity of our work with other 3D printing and traditional methods. Figure S12: setup for the test of thermal control structures with 3D printed shapes. Figure S13: flame-retardant test of natural nacre. Figure S14: TGA tests of pure SI, BNs, and SI/55 wt% BNs and the [file 9840574.f1.zip › Supplemetal Figures/Figure S1.png]

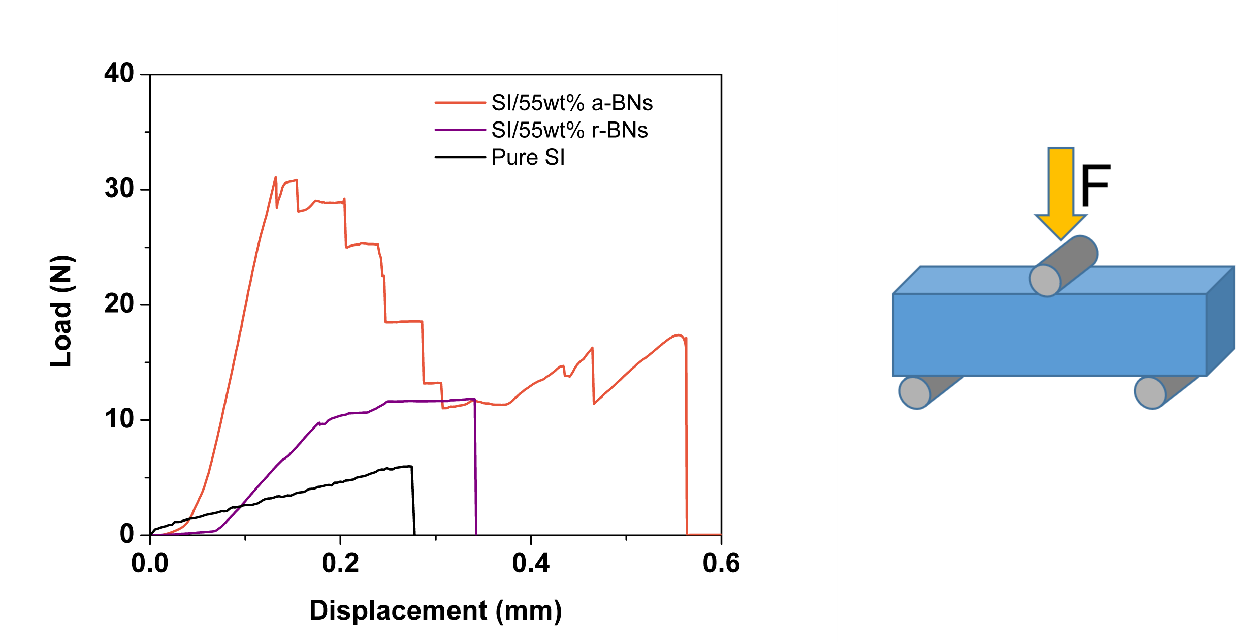

Supplement: Supplementary Materials — Table S1: comparison of mechanical protection property and shape complexity for boron nitride nanoplatelets (BNs) and nacre-inspired flame-retardant structures. Figure S1: SEM images of aligned BNs and magnified view of BNs. Figure S2: (a) representation of the surface modification procedure of BNs by 3-(trimethoxysilyl)propyl methacrylate (TMSPMA). (b) Schematic diagram shows the alignment of BNs in photocurable monomer and the covalent bonding between TMSPMA and photocurable monomer. Figure S3: FTIR spectrum of pure BNs and surface modified BNs by 3-(trimethoxysilyl)propyl methacrylate (TMSPMA). The diagram on the right shows the corresponding chemical bonding on the FTIR spectrum. Figure S4: SEM images of the original BNs (unmodified) and the TMSPMA-grafted BNs. Comparison of stress distribution during the sliding of adjacent BNs for the unmodified BNs and the TMSPMA-grafted BNs simulated by COMSOL Multiphysics. Figure S5: study of the efficiency of alignment of BNs with the gap between the doctor blade and the substrate, (a) 100 μm, (b) 300 μm,and (c) 500 μm. Figure S6: SEM images of SI/rBNs, SI/a-BNs with the unmodified BNs, and SI/a-BNs with the TMSPMA-grafted BNs. Figure S7: changes of cure depth with the fraction of BNs. Figure S8: comparison of 3-point-bending tests for 3D printed a-BNs with the unmodified BNs and the TMSPMA-grafted BNs. Figure S9: crack deflection, a-BN bridging, and pulling out for 3D printed nacre-inspired structures with TMSPMA grafted a-BNs. Figure S10: the standard three-point-bending tests were performed to study the flexural strength of the 3D-printed structures. Figure S11: compression test of the 3D printed nacre with aligned BNs. Table S2: comparison of thermal conductivity of our work with other 3D printing and traditional methods. Figure S12: setup for the test of thermal control structures with 3D printed shapes. Figure S13: flame-retardant test of natural nacre. Figure S14: TGA tests of pure SI, BNs, and SI/55 wt% BNs and the [file 9840574.f1.zip › Supplemetal Figures/Figure S10.png]

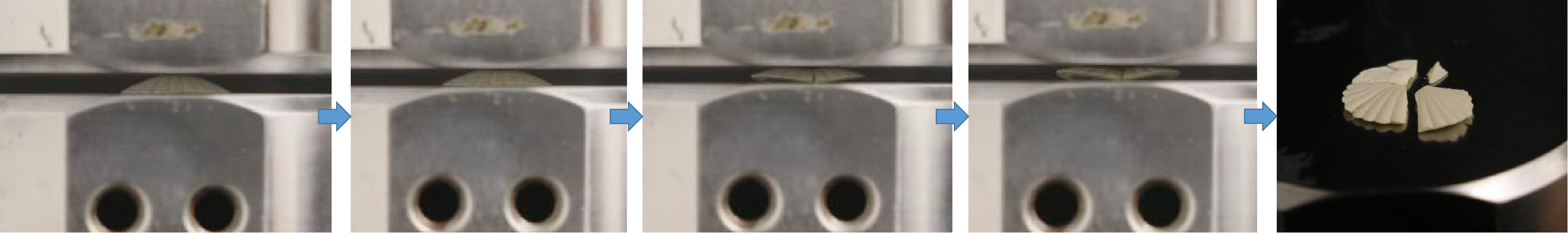

Supplement: Supplementary Materials — Table S1: comparison of mechanical protection property and shape complexity for boron nitride nanoplatelets (BNs) and nacre-inspired flame-retardant structures. Figure S1: SEM images of aligned BNs and magnified view of BNs. Figure S2: (a) representation of the surface modification procedure of BNs by 3-(trimethoxysilyl)propyl methacrylate (TMSPMA). (b) Schematic diagram shows the alignment of BNs in photocurable monomer and the covalent bonding between TMSPMA and photocurable monomer. Figure S3: FTIR spectrum of pure BNs and surface modified BNs by 3-(trimethoxysilyl)propyl methacrylate (TMSPMA). The diagram on the right shows the corresponding chemical bonding on the FTIR spectrum. Figure S4: SEM images of the original BNs (unmodified) and the TMSPMA-grafted BNs. Comparison of stress distribution during the sliding of adjacent BNs for the unmodified BNs and the TMSPMA-grafted BNs simulated by COMSOL Multiphysics. Figure S5: study of the efficiency of alignment of BNs with the gap between the doctor blade and the substrate, (a) 100 μm, (b) 300 μm,and (c) 500 μm. Figure S6: SEM images of SI/rBNs, SI/a-BNs with the unmodified BNs, and SI/a-BNs with the TMSPMA-grafted BNs. Figure S7: changes of cure depth with the fraction of BNs. Figure S8: comparison of 3-point-bending tests for 3D printed a-BNs with the unmodified BNs and the TMSPMA-grafted BNs. Figure S9: crack deflection, a-BN bridging, and pulling out for 3D printed nacre-inspired structures with TMSPMA grafted a-BNs. Figure S10: the standard three-point-bending tests were performed to study the flexural strength of the 3D-printed structures. Figure S11: compression test of the 3D printed nacre with aligned BNs. Table S2: comparison of thermal conductivity of our work with other 3D printing and traditional methods. Figure S12: setup for the test of thermal control structures with 3D printed shapes. Figure S13: flame-retardant test of natural nacre. Figure S14: TGA tests of pure SI, BNs, and SI/55 wt% BNs and the [file 9840574.f1.zip › Supplemetal Figures/Figure S11.png]

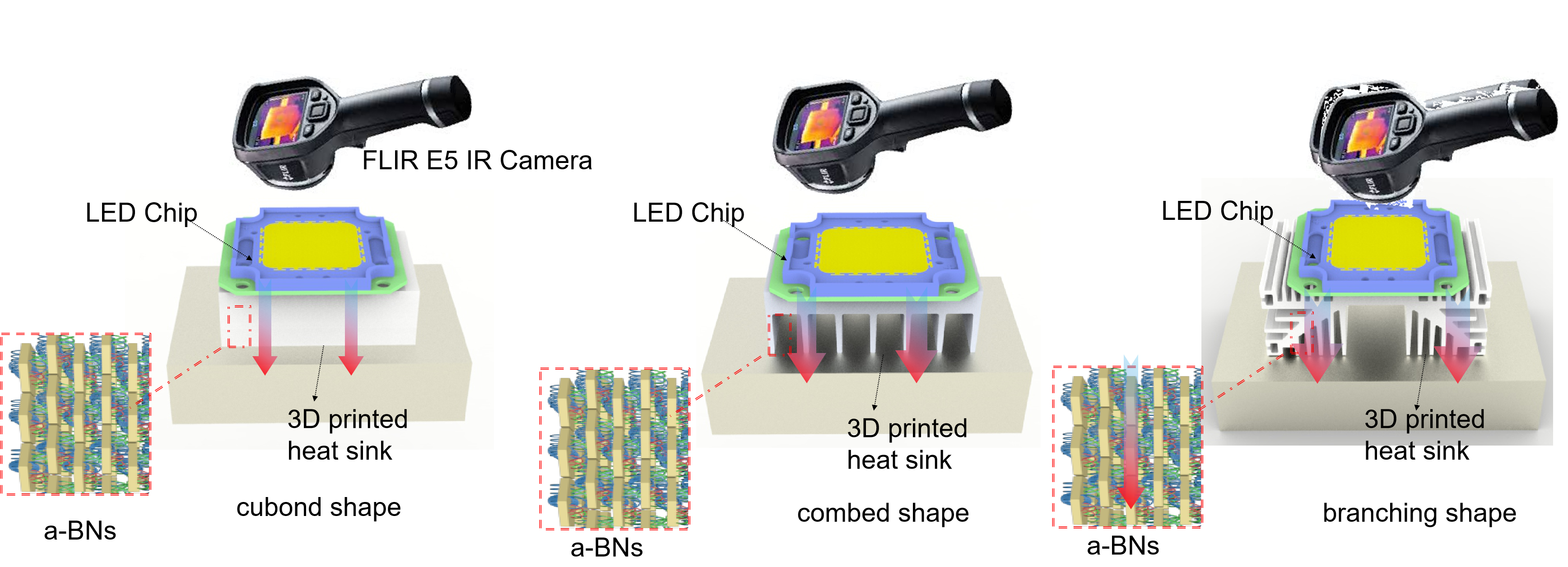

Supplement: Supplementary Materials — Table S1: comparison of mechanical protection property and shape complexity for boron nitride nanoplatelets (BNs) and nacre-inspired flame-retardant structures. Figure S1: SEM images of aligned BNs and magnified view of BNs. Figure S2: (a) representation of the surface modification procedure of BNs by 3-(trimethoxysilyl)propyl methacrylate (TMSPMA). (b) Schematic diagram shows the alignment of BNs in photocurable monomer and the covalent bonding between TMSPMA and photocurable monomer. Figure S3: FTIR spectrum of pure BNs and surface modified BNs by 3-(trimethoxysilyl)propyl methacrylate (TMSPMA). The diagram on the right shows the corresponding chemical bonding on the FTIR spectrum. Figure S4: SEM images of the original BNs (unmodified) and the TMSPMA-grafted BNs. Comparison of stress distribution during the sliding of adjacent BNs for the unmodified BNs and the TMSPMA-grafted BNs simulated by COMSOL Multiphysics. Figure S5: study of the efficiency of alignment of BNs with the gap between the doctor blade and the substrate, (a) 100 μm, (b) 300 μm,and (c) 500 μm. Figure S6: SEM images of SI/rBNs, SI/a-BNs with the unmodified BNs, and SI/a-BNs with the TMSPMA-grafted BNs. Figure S7: changes of cure depth with the fraction of BNs. Figure S8: comparison of 3-point-bending tests for 3D printed a-BNs with the unmodified BNs and the TMSPMA-grafted BNs. Figure S9: crack deflection, a-BN bridging, and pulling out for 3D printed nacre-inspired structures with TMSPMA grafted a-BNs. Figure S10: the standard three-point-bending tests were performed to study the flexural strength of the 3D-printed structures. Figure S11: compression test of the 3D printed nacre with aligned BNs. Table S2: comparison of thermal conductivity of our work with other 3D printing and traditional methods. Figure S12: setup for the test of thermal control structures with 3D printed shapes. Figure S13: flame-retardant test of natural nacre. Figure S14: TGA tests of pure SI, BNs, and SI/55 wt% BNs and the [file 9840574.f1.zip › Supplemetal Figures/Figure S12.png]

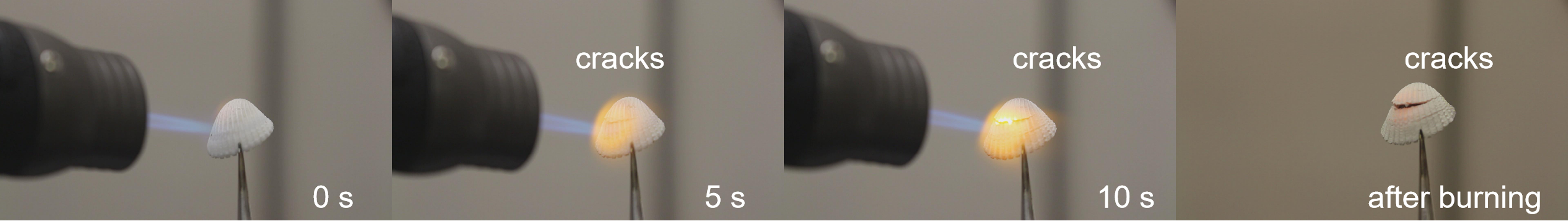

Supplement: Supplementary Materials — Table S1: comparison of mechanical protection property and shape complexity for boron nitride nanoplatelets (BNs) and nacre-inspired flame-retardant structures. Figure S1: SEM images of aligned BNs and magnified view of BNs. Figure S2: (a) representation of the surface modification procedure of BNs by 3-(trimethoxysilyl)propyl methacrylate (TMSPMA). (b) Schematic diagram shows the alignment of BNs in photocurable monomer and the covalent bonding between TMSPMA and photocurable monomer. Figure S3: FTIR spectrum of pure BNs and surface modified BNs by 3-(trimethoxysilyl)propyl methacrylate (TMSPMA). The diagram on the right shows the corresponding chemical bonding on the FTIR spectrum. Figure S4: SEM images of the original BNs (unmodified) and the TMSPMA-grafted BNs. Comparison of stress distribution during the sliding of adjacent BNs for the unmodified BNs and the TMSPMA-grafted BNs simulated by COMSOL Multiphysics. Figure S5: study of the efficiency of alignment of BNs with the gap between the doctor blade and the substrate, (a) 100 μm, (b) 300 μm,and (c) 500 μm. Figure S6: SEM images of SI/rBNs, SI/a-BNs with the unmodified BNs, and SI/a-BNs with the TMSPMA-grafted BNs. Figure S7: changes of cure depth with the fraction of BNs. Figure S8: comparison of 3-point-bending tests for 3D printed a-BNs with the unmodified BNs and the TMSPMA-grafted BNs. Figure S9: crack deflection, a-BN bridging, and pulling out for 3D printed nacre-inspired structures with TMSPMA grafted a-BNs. Figure S10: the standard three-point-bending tests were performed to study the flexural strength of the 3D-printed structures. Figure S11: compression test of the 3D printed nacre with aligned BNs. Table S2: comparison of thermal conductivity of our work with other 3D printing and traditional methods. Figure S12: setup for the test of thermal control structures with 3D printed shapes. Figure S13: flame-retardant test of natural nacre. Figure S14: TGA tests of pure SI, BNs, and SI/55 wt% BNs and the [file 9840574.f1.zip › Supplemetal Figures/Figure S13.png]

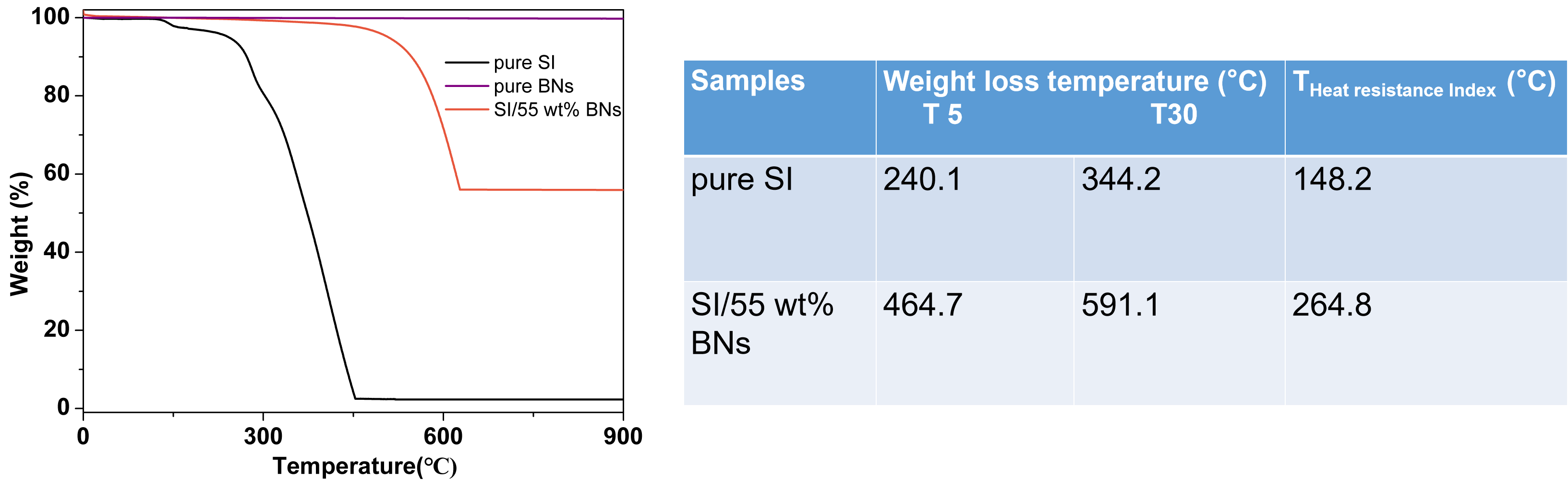

Supplement: Supplementary Materials — Table S1: comparison of mechanical protection property and shape complexity for boron nitride nanoplatelets (BNs) and nacre-inspired flame-retardant structures. Figure S1: SEM images of aligned BNs and magnified view of BNs. Figure S2: (a) representation of the surface modification procedure of BNs by 3-(trimethoxysilyl)propyl methacrylate (TMSPMA). (b) Schematic diagram shows the alignment of BNs in photocurable monomer and the covalent bonding between TMSPMA and photocurable monomer. Figure S3: FTIR spectrum of pure BNs and surface modified BNs by 3-(trimethoxysilyl)propyl methacrylate (TMSPMA). The diagram on the right shows the corresponding chemical bonding on the FTIR spectrum. Figure S4: SEM images of the original BNs (unmodified) and the TMSPMA-grafted BNs. Comparison of stress distribution during the sliding of adjacent BNs for the unmodified BNs and the TMSPMA-grafted BNs simulated by COMSOL Multiphysics. Figure S5: study of the efficiency of alignment of BNs with the gap between the doctor blade and the substrate, (a) 100 μm, (b) 300 μm,and (c) 500 μm. Figure S6: SEM images of SI/rBNs, SI/a-BNs with the unmodified BNs, and SI/a-BNs with the TMSPMA-grafted BNs. Figure S7: changes of cure depth with the fraction of BNs. Figure S8: comparison of 3-point-bending tests for 3D printed a-BNs with the unmodified BNs and the TMSPMA-grafted BNs. Figure S9: crack deflection, a-BN bridging, and pulling out for 3D printed nacre-inspired structures with TMSPMA grafted a-BNs. Figure S10: the standard three-point-bending tests were performed to study the flexural strength of the 3D-printed structures. Figure S11: compression test of the 3D printed nacre with aligned BNs. Table S2: comparison of thermal conductivity of our work with other 3D printing and traditional methods. Figure S12: setup for the test of thermal control structures with 3D printed shapes. Figure S13: flame-retardant test of natural nacre. Figure S14: TGA tests of pure SI, BNs, and SI/55 wt% BNs and the [file 9840574.f1.zip › Supplemetal Figures/Figure S14.png]

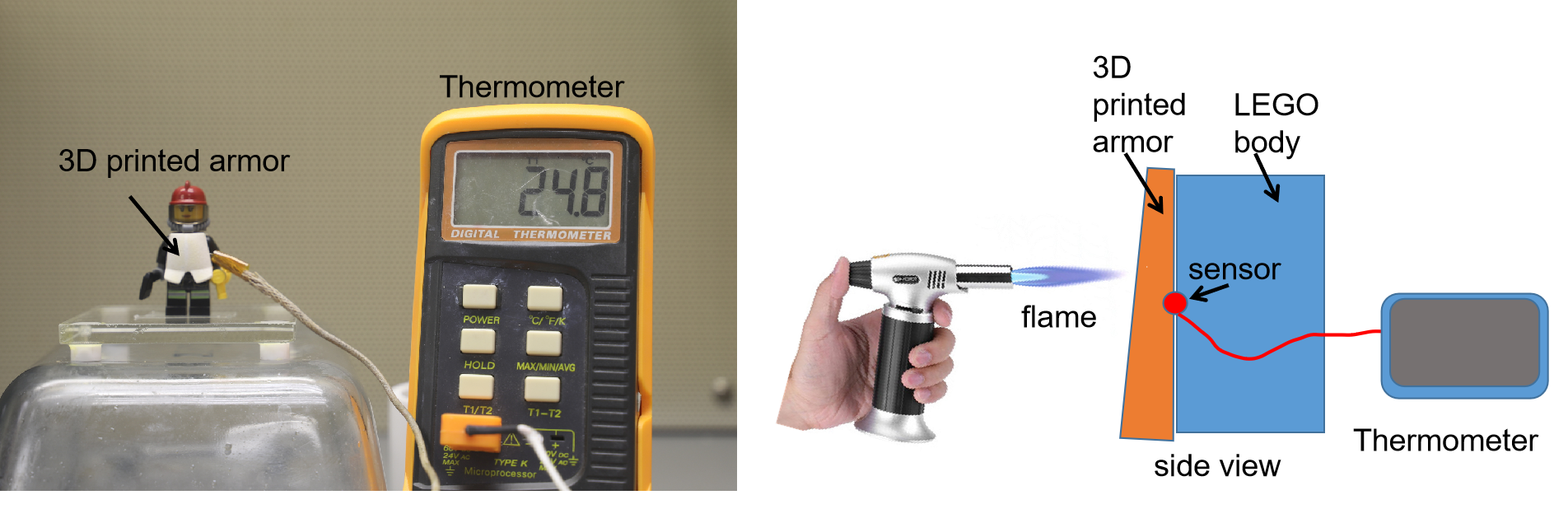

Supplement: Supplementary Materials — Table S1: comparison of mechanical protection property and shape complexity for boron nitride nanoplatelets (BNs) and nacre-inspired flame-retardant structures. Figure S1: SEM images of aligned BNs and magnified view of BNs. Figure S2: (a) representation of the surface modification procedure of BNs by 3-(trimethoxysilyl)propyl methacrylate (TMSPMA). (b) Schematic diagram shows the alignment of BNs in photocurable monomer and the covalent bonding between TMSPMA and photocurable monomer. Figure S3: FTIR spectrum of pure BNs and surface modified BNs by 3-(trimethoxysilyl)propyl methacrylate (TMSPMA). The diagram on the right shows the corresponding chemical bonding on the FTIR spectrum. Figure S4: SEM images of the original BNs (unmodified) and the TMSPMA-grafted BNs. Comparison of stress distribution during the sliding of adjacent BNs for the unmodified BNs and the TMSPMA-grafted BNs simulated by COMSOL Multiphysics. Figure S5: study of the efficiency of alignment of BNs with the gap between the doctor blade and the substrate, (a) 100 μm, (b) 300 μm,and (c) 500 μm. Figure S6: SEM images of SI/rBNs, SI/a-BNs with the unmodified BNs, and SI/a-BNs with the TMSPMA-grafted BNs. Figure S7: changes of cure depth with the fraction of BNs. Figure S8: comparison of 3-point-bending tests for 3D printed a-BNs with the unmodified BNs and the TMSPMA-grafted BNs. Figure S9: crack deflection, a-BN bridging, and pulling out for 3D printed nacre-inspired structures with TMSPMA grafted a-BNs. Figure S10: the standard three-point-bending tests were performed to study the flexural strength of the 3D-printed structures. Figure S11: compression test of the 3D printed nacre with aligned BNs. Table S2: comparison of thermal conductivity of our work with other 3D printing and traditional methods. Figure S12: setup for the test of thermal control structures with 3D printed shapes. Figure S13: flame-retardant test of natural nacre. Figure S14: TGA tests of pure SI, BNs, and SI/55 wt% BNs and the [file 9840574.f1.zip › Supplemetal Figures/Figure S15.png]

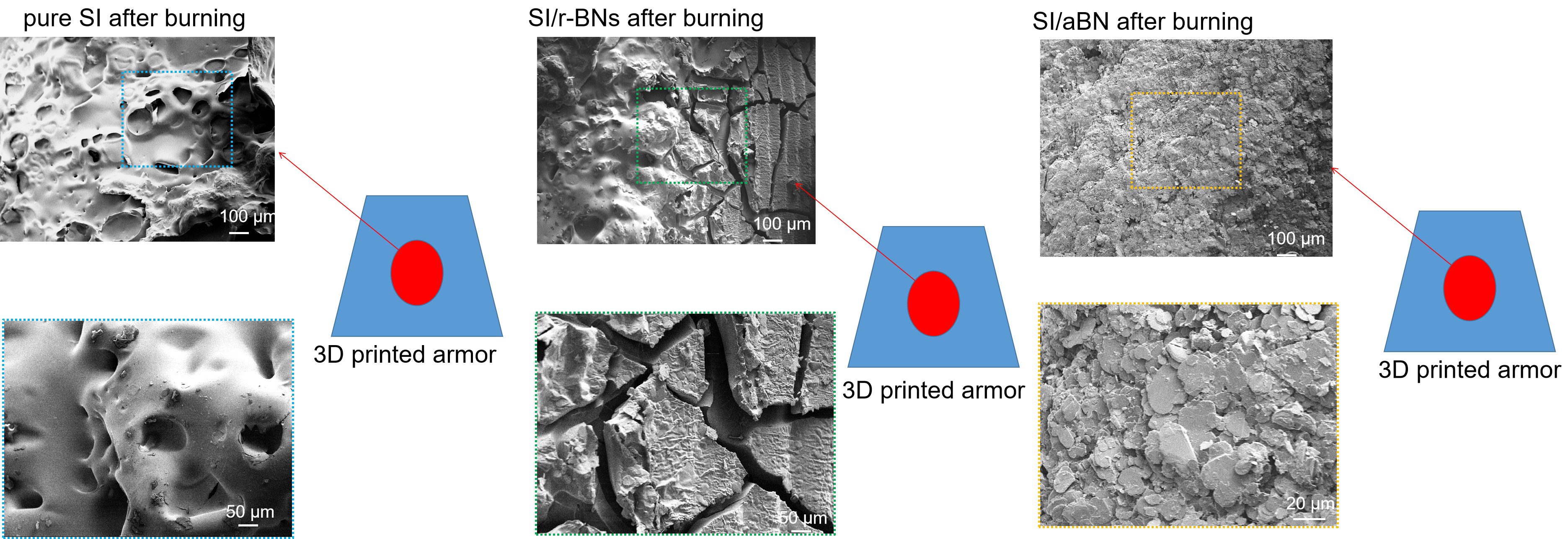

Supplement: Supplementary Materials — Table S1: comparison of mechanical protection property and shape complexity for boron nitride nanoplatelets (BNs) and nacre-inspired flame-retardant structures. Figure S1: SEM images of aligned BNs and magnified view of BNs. Figure S2: (a) representation of the surface modification procedure of BNs by 3-(trimethoxysilyl)propyl methacrylate (TMSPMA). (b) Schematic diagram shows the alignment of BNs in photocurable monomer and the covalent bonding between TMSPMA and photocurable monomer. Figure S3: FTIR spectrum of pure BNs and surface modified BNs by 3-(trimethoxysilyl)propyl methacrylate (TMSPMA). The diagram on the right shows the corresponding chemical bonding on the FTIR spectrum. Figure S4: SEM images of the original BNs (unmodified) and the TMSPMA-grafted BNs. Comparison of stress distribution during the sliding of adjacent BNs for the unmodified BNs and the TMSPMA-grafted BNs simulated by COMSOL Multiphysics. Figure S5: study of the efficiency of alignment of BNs with the gap between the doctor blade and the substrate, (a) 100 μm, (b) 300 μm,and (c) 500 μm. Figure S6: SEM images of SI/rBNs, SI/a-BNs with the unmodified BNs, and SI/a-BNs with the TMSPMA-grafted BNs. Figure S7: changes of cure depth with the fraction of BNs. Figure S8: comparison of 3-point-bending tests for 3D printed a-BNs with the unmodified BNs and the TMSPMA-grafted BNs. Figure S9: crack deflection, a-BN bridging, and pulling out for 3D printed nacre-inspired structures with TMSPMA grafted a-BNs. Figure S10: the standard three-point-bending tests were performed to study the flexural strength of the 3D-printed structures. Figure S11: compression test of the 3D printed nacre with aligned BNs. Table S2: comparison of thermal conductivity of our work with other 3D printing and traditional methods. Figure S12: setup for the test of thermal control structures with 3D printed shapes. Figure S13: flame-retardant test of natural nacre. Figure S14: TGA tests of pure SI, BNs, and SI/55 wt% BNs and the [file 9840574.f1.zip › Supplemetal Figures/Figure S16.png]

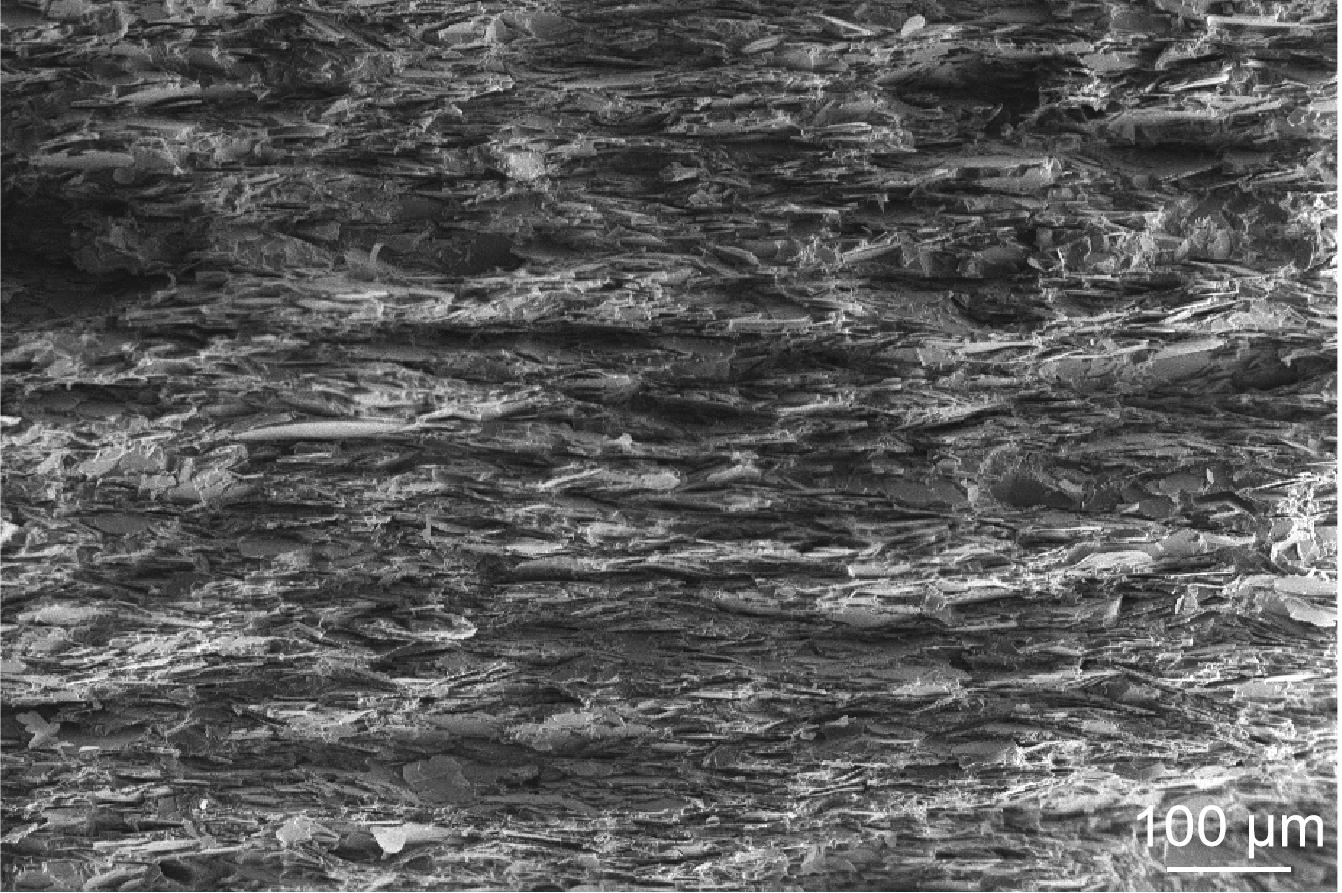

Supplement: Supplementary Materials — Table S1: comparison of mechanical protection property and shape complexity for boron nitride nanoplatelets (BNs) and nacre-inspired flame-retardant structures. Figure S1: SEM images of aligned BNs and magnified view of BNs. Figure S2: (a) representation of the surface modification procedure of BNs by 3-(trimethoxysilyl)propyl methacrylate (TMSPMA). (b) Schematic diagram shows the alignment of BNs in photocurable monomer and the covalent bonding between TMSPMA and photocurable monomer. Figure S3: FTIR spectrum of pure BNs and surface modified BNs by 3-(trimethoxysilyl)propyl methacrylate (TMSPMA). The diagram on the right shows the corresponding chemical bonding on the FTIR spectrum. Figure S4: SEM images of the original BNs (unmodified) and the TMSPMA-grafted BNs. Comparison of stress distribution during the sliding of adjacent BNs for the unmodified BNs and the TMSPMA-grafted BNs simulated by COMSOL Multiphysics. Figure S5: study of the efficiency of alignment of BNs with the gap between the doctor blade and the substrate, (a) 100 μm, (b) 300 μm,and (c) 500 μm. Figure S6: SEM images of SI/rBNs, SI/a-BNs with the unmodified BNs, and SI/a-BNs with the TMSPMA-grafted BNs. Figure S7: changes of cure depth with the fraction of BNs. Figure S8: comparison of 3-point-bending tests for 3D printed a-BNs with the unmodified BNs and the TMSPMA-grafted BNs. Figure S9: crack deflection, a-BN bridging, and pulling out for 3D printed nacre-inspired structures with TMSPMA grafted a-BNs. Figure S10: the standard three-point-bending tests were performed to study the flexural strength of the 3D-printed structures. Figure S11: compression test of the 3D printed nacre with aligned BNs. Table S2: comparison of thermal conductivity of our work with other 3D printing and traditional methods. Figure S12: setup for the test of thermal control structures with 3D printed shapes. Figure S13: flame-retardant test of natural nacre. Figure S14: TGA tests of pure SI, BNs, and SI/55 wt% BNs and the [file 9840574.f1.zip › Supplemetal Figures/Figure S17.png]

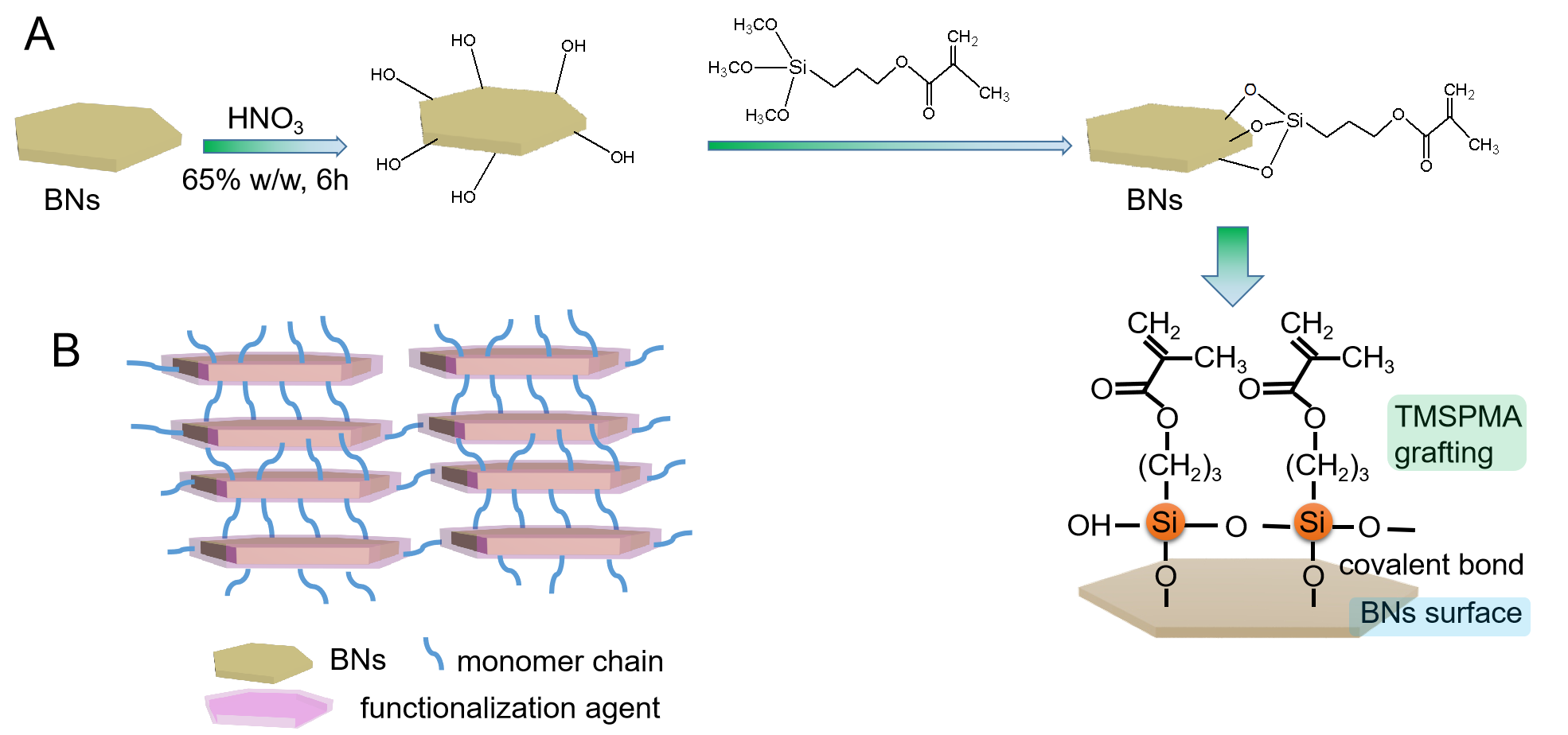

Supplement: Supplementary Materials — Table S1: comparison of mechanical protection property and shape complexity for boron nitride nanoplatelets (BNs) and nacre-inspired flame-retardant structures. Figure S1: SEM images of aligned BNs and magnified view of BNs. Figure S2: (a) representation of the surface modification procedure of BNs by 3-(trimethoxysilyl)propyl methacrylate (TMSPMA). (b) Schematic diagram shows the alignment of BNs in photocurable monomer and the covalent bonding between TMSPMA and photocurable monomer. Figure S3: FTIR spectrum of pure BNs and surface modified BNs by 3-(trimethoxysilyl)propyl methacrylate (TMSPMA). The diagram on the right shows the corresponding chemical bonding on the FTIR spectrum. Figure S4: SEM images of the original BNs (unmodified) and the TMSPMA-grafted BNs. Comparison of stress distribution during the sliding of adjacent BNs for the unmodified BNs and the TMSPMA-grafted BNs simulated by COMSOL Multiphysics. Figure S5: study of the efficiency of alignment of BNs with the gap between the doctor blade and the substrate, (a) 100 μm, (b) 300 μm,and (c) 500 μm. Figure S6: SEM images of SI/rBNs, SI/a-BNs with the unmodified BNs, and SI/a-BNs with the TMSPMA-grafted BNs. Figure S7: changes of cure depth with the fraction of BNs. Figure S8: comparison of 3-point-bending tests for 3D printed a-BNs with the unmodified BNs and the TMSPMA-grafted BNs. Figure S9: crack deflection, a-BN bridging, and pulling out for 3D printed nacre-inspired structures with TMSPMA grafted a-BNs. Figure S10: the standard three-point-bending tests were performed to study the flexural strength of the 3D-printed structures. Figure S11: compression test of the 3D printed nacre with aligned BNs. Table S2: comparison of thermal conductivity of our work with other 3D printing and traditional methods. Figure S12: setup for the test of thermal control structures with 3D printed shapes. Figure S13: flame-retardant test of natural nacre. Figure S14: TGA tests of pure SI, BNs, and SI/55 wt% BNs and the [file 9840574.f1.zip › Supplemetal Figures/Figure S2.png]

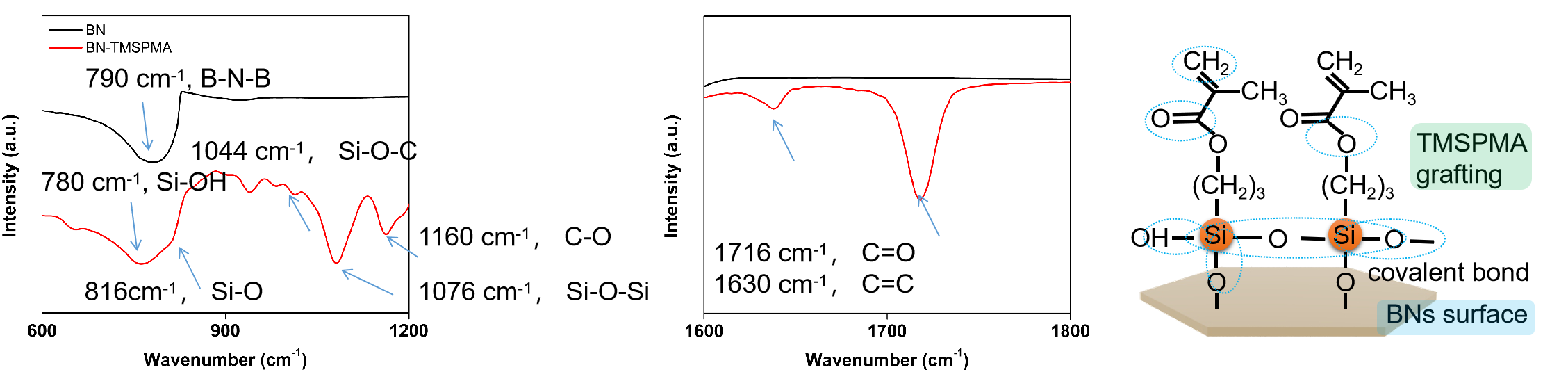

Supplement: Supplementary Materials — Table S1: comparison of mechanical protection property and shape complexity for boron nitride nanoplatelets (BNs) and nacre-inspired flame-retardant structures. Figure S1: SEM images of aligned BNs and magnified view of BNs. Figure S2: (a) representation of the surface modification procedure of BNs by 3-(trimethoxysilyl)propyl methacrylate (TMSPMA). (b) Schematic diagram shows the alignment of BNs in photocurable monomer and the covalent bonding between TMSPMA and photocurable monomer. Figure S3: FTIR spectrum of pure BNs and surface modified BNs by 3-(trimethoxysilyl)propyl methacrylate (TMSPMA). The diagram on the right shows the corresponding chemical bonding on the FTIR spectrum. Figure S4: SEM images of the original BNs (unmodified) and the TMSPMA-grafted BNs. Comparison of stress distribution during the sliding of adjacent BNs for the unmodified BNs and the TMSPMA-grafted BNs simulated by COMSOL Multiphysics. Figure S5: study of the efficiency of alignment of BNs with the gap between the doctor blade and the substrate, (a) 100 μm, (b) 300 μm,and (c) 500 μm. Figure S6: SEM images of SI/rBNs, SI/a-BNs with the unmodified BNs, and SI/a-BNs with the TMSPMA-grafted BNs. Figure S7: changes of cure depth with the fraction of BNs. Figure S8: comparison of 3-point-bending tests for 3D printed a-BNs with the unmodified BNs and the TMSPMA-grafted BNs. Figure S9: crack deflection, a-BN bridging, and pulling out for 3D printed nacre-inspired structures with TMSPMA grafted a-BNs. Figure S10: the standard three-point-bending tests were performed to study the flexural strength of the 3D-printed structures. Figure S11: compression test of the 3D printed nacre with aligned BNs. Table S2: comparison of thermal conductivity of our work with other 3D printing and traditional methods. Figure S12: setup for the test of thermal control structures with 3D printed shapes. Figure S13: flame-retardant test of natural nacre. Figure S14: TGA tests of pure SI, BNs, and SI/55 wt% BNs and the [file 9840574.f1.zip › Supplemetal Figures/Figure S3.png]

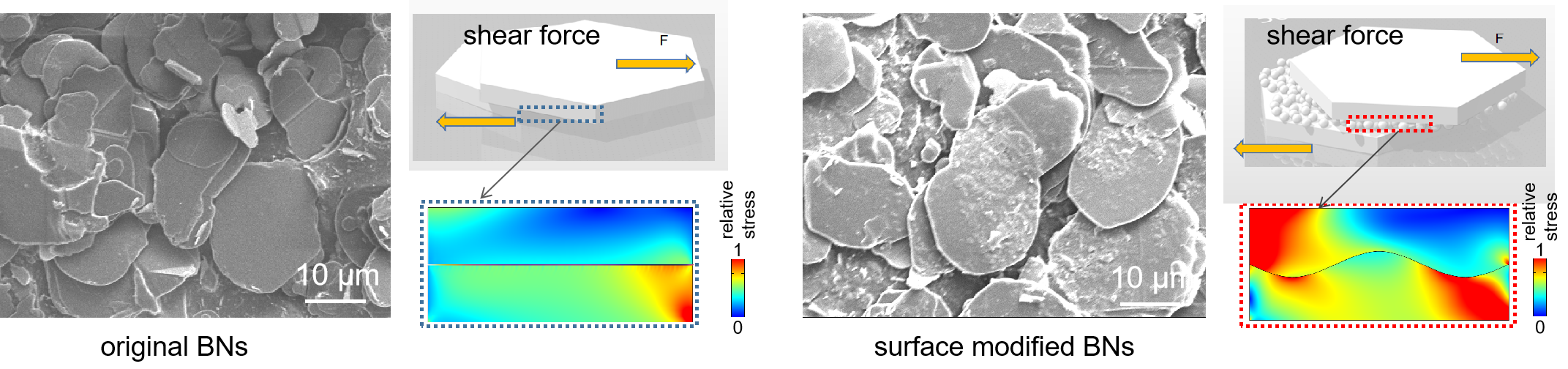

Supplement: Supplementary Materials — Table S1: comparison of mechanical protection property and shape complexity for boron nitride nanoplatelets (BNs) and nacre-inspired flame-retardant structures. Figure S1: SEM images of aligned BNs and magnified view of BNs. Figure S2: (a) representation of the surface modification procedure of BNs by 3-(trimethoxysilyl)propyl methacrylate (TMSPMA). (b) Schematic diagram shows the alignment of BNs in photocurable monomer and the covalent bonding between TMSPMA and photocurable monomer. Figure S3: FTIR spectrum of pure BNs and surface modified BNs by 3-(trimethoxysilyl)propyl methacrylate (TMSPMA). The diagram on the right shows the corresponding chemical bonding on the FTIR spectrum. Figure S4: SEM images of the original BNs (unmodified) and the TMSPMA-grafted BNs. Comparison of stress distribution during the sliding of adjacent BNs for the unmodified BNs and the TMSPMA-grafted BNs simulated by COMSOL Multiphysics. Figure S5: study of the efficiency of alignment of BNs with the gap between the doctor blade and the substrate, (a) 100 μm, (b) 300 μm,and (c) 500 μm. Figure S6: SEM images of SI/rBNs, SI/a-BNs with the unmodified BNs, and SI/a-BNs with the TMSPMA-grafted BNs. Figure S7: changes of cure depth with the fraction of BNs. Figure S8: comparison of 3-point-bending tests for 3D printed a-BNs with the unmodified BNs and the TMSPMA-grafted BNs. Figure S9: crack deflection, a-BN bridging, and pulling out for 3D printed nacre-inspired structures with TMSPMA grafted a-BNs. Figure S10: the standard three-point-bending tests were performed to study the flexural strength of the 3D-printed structures. Figure S11: compression test of the 3D printed nacre with aligned BNs. Table S2: comparison of thermal conductivity of our work with other 3D printing and traditional methods. Figure S12: setup for the test of thermal control structures with 3D printed shapes. Figure S13: flame-retardant test of natural nacre. Figure S14: TGA tests of pure SI, BNs, and SI/55 wt% BNs and the [file 9840574.f1.zip › Supplemetal Figures/Figure S4.png]

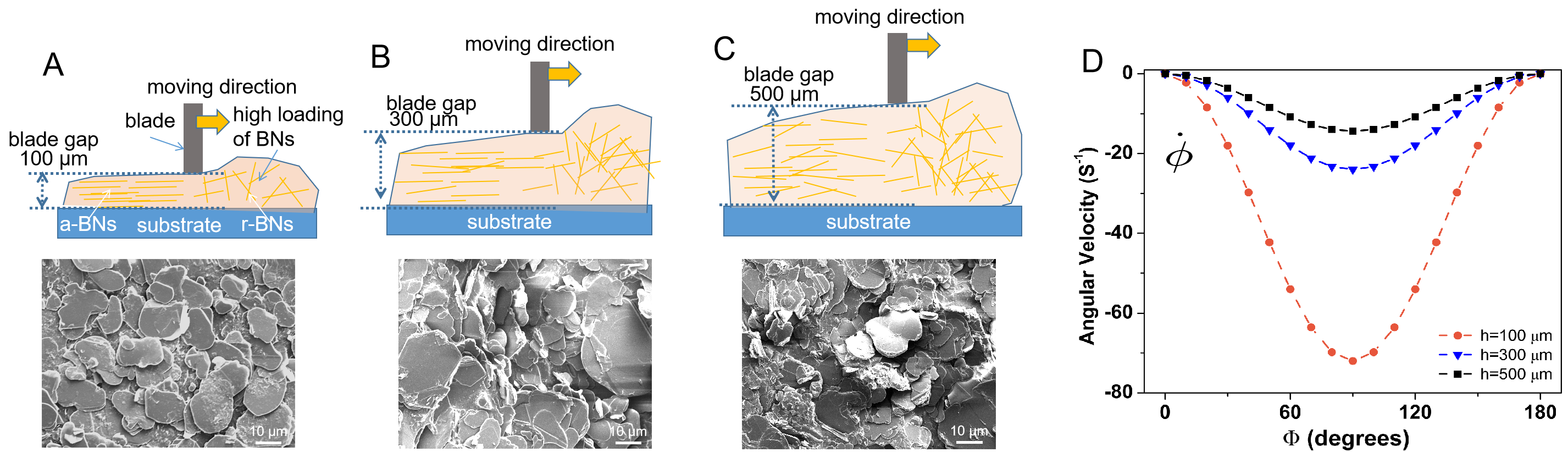

Supplement: Supplementary Materials — Table S1: comparison of mechanical protection property and shape complexity for boron nitride nanoplatelets (BNs) and nacre-inspired flame-retardant structures. Figure S1: SEM images of aligned BNs and magnified view of BNs. Figure S2: (a) representation of the surface modification procedure of BNs by 3-(trimethoxysilyl)propyl methacrylate (TMSPMA). (b) Schematic diagram shows the alignment of BNs in photocurable monomer and the covalent bonding between TMSPMA and photocurable monomer. Figure S3: FTIR spectrum of pure BNs and surface modified BNs by 3-(trimethoxysilyl)propyl methacrylate (TMSPMA). The diagram on the right shows the corresponding chemical bonding on the FTIR spectrum. Figure S4: SEM images of the original BNs (unmodified) and the TMSPMA-grafted BNs. Comparison of stress distribution during the sliding of adjacent BNs for the unmodified BNs and the TMSPMA-grafted BNs simulated by COMSOL Multiphysics. Figure S5: study of the efficiency of alignment of BNs with the gap between the doctor blade and the substrate, (a) 100 μm, (b) 300 μm,and (c) 500 μm. Figure S6: SEM images of SI/rBNs, SI/a-BNs with the unmodified BNs, and SI/a-BNs with the TMSPMA-grafted BNs. Figure S7: changes of cure depth with the fraction of BNs. Figure S8: comparison of 3-point-bending tests for 3D printed a-BNs with the unmodified BNs and the TMSPMA-grafted BNs. Figure S9: crack deflection, a-BN bridging, and pulling out for 3D printed nacre-inspired structures with TMSPMA grafted a-BNs. Figure S10: the standard three-point-bending tests were performed to study the flexural strength of the 3D-printed structures. Figure S11: compression test of the 3D printed nacre with aligned BNs. Table S2: comparison of thermal conductivity of our work with other 3D printing and traditional methods. Figure S12: setup for the test of thermal control structures with 3D printed shapes. Figure S13: flame-retardant test of natural nacre. Figure S14: TGA tests of pure SI, BNs, and SI/55 wt% BNs and the [file 9840574.f1.zip › Supplemetal Figures/Figure S5.png]

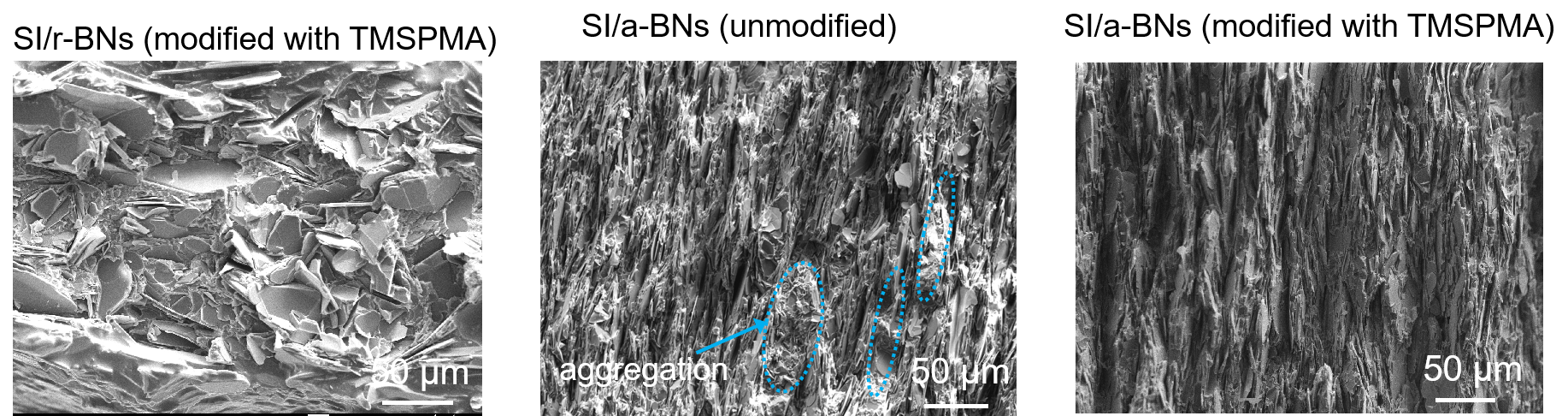

Supplement: Supplementary Materials — Table S1: comparison of mechanical protection property and shape complexity for boron nitride nanoplatelets (BNs) and nacre-inspired flame-retardant structures. Figure S1: SEM images of aligned BNs and magnified view of BNs. Figure S2: (a) representation of the surface modification procedure of BNs by 3-(trimethoxysilyl)propyl methacrylate (TMSPMA). (b) Schematic diagram shows the alignment of BNs in photocurable monomer and the covalent bonding between TMSPMA and photocurable monomer. Figure S3: FTIR spectrum of pure BNs and surface modified BNs by 3-(trimethoxysilyl)propyl methacrylate (TMSPMA). The diagram on the right shows the corresponding chemical bonding on the FTIR spectrum. Figure S4: SEM images of the original BNs (unmodified) and the TMSPMA-grafted BNs. Comparison of stress distribution during the sliding of adjacent BNs for the unmodified BNs and the TMSPMA-grafted BNs simulated by COMSOL Multiphysics. Figure S5: study of the efficiency of alignment of BNs with the gap between the doctor blade and the substrate, (a) 100 μm, (b) 300 μm,and (c) 500 μm. Figure S6: SEM images of SI/rBNs, SI/a-BNs with the unmodified BNs, and SI/a-BNs with the TMSPMA-grafted BNs. Figure S7: changes of cure depth with the fraction of BNs. Figure S8: comparison of 3-point-bending tests for 3D printed a-BNs with the unmodified BNs and the TMSPMA-grafted BNs. Figure S9: crack deflection, a-BN bridging, and pulling out for 3D printed nacre-inspired structures with TMSPMA grafted a-BNs. Figure S10: the standard three-point-bending tests were performed to study the flexural strength of the 3D-printed structures. Figure S11: compression test of the 3D printed nacre with aligned BNs. Table S2: comparison of thermal conductivity of our work with other 3D printing and traditional methods. Figure S12: setup for the test of thermal control structures with 3D printed shapes. Figure S13: flame-retardant test of natural nacre. Figure S14: TGA tests of pure SI, BNs, and SI/55 wt% BNs and the [file 9840574.f1.zip › Supplemetal Figures/Figure S6.png]

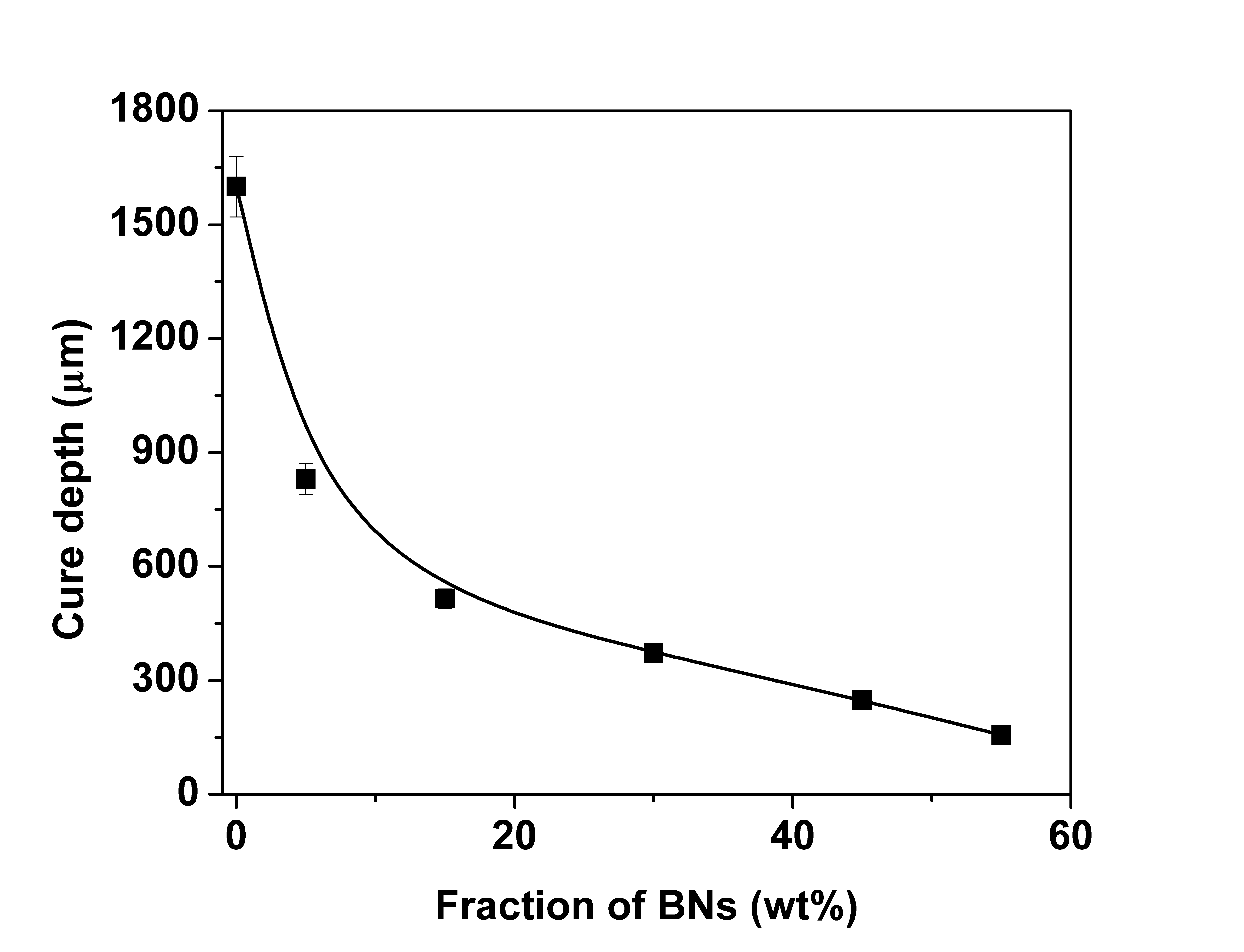

Supplement: Supplementary Materials — Table S1: comparison of mechanical protection property and shape complexity for boron nitride nanoplatelets (BNs) and nacre-inspired flame-retardant structures. Figure S1: SEM images of aligned BNs and magnified view of BNs. Figure S2: (a) representation of the surface modification procedure of BNs by 3-(trimethoxysilyl)propyl methacrylate (TMSPMA). (b) Schematic diagram shows the alignment of BNs in photocurable monomer and the covalent bonding between TMSPMA and photocurable monomer. Figure S3: FTIR spectrum of pure BNs and surface modified BNs by 3-(trimethoxysilyl)propyl methacrylate (TMSPMA). The diagram on the right shows the corresponding chemical bonding on the FTIR spectrum. Figure S4: SEM images of the original BNs (unmodified) and the TMSPMA-grafted BNs. Comparison of stress distribution during the sliding of adjacent BNs for the unmodified BNs and the TMSPMA-grafted BNs simulated by COMSOL Multiphysics. Figure S5: study of the efficiency of alignment of BNs with the gap between the doctor blade and the substrate, (a) 100 μm, (b) 300 μm,and (c) 500 μm. Figure S6: SEM images of SI/rBNs, SI/a-BNs with the unmodified BNs, and SI/a-BNs with the TMSPMA-grafted BNs. Figure S7: changes of cure depth with the fraction of BNs. Figure S8: comparison of 3-point-bending tests for 3D printed a-BNs with the unmodified BNs and the TMSPMA-grafted BNs. Figure S9: crack deflection, a-BN bridging, and pulling out for 3D printed nacre-inspired structures with TMSPMA grafted a-BNs. Figure S10: the standard three-point-bending tests were performed to study the flexural strength of the 3D-printed structures. Figure S11: compression test of the 3D printed nacre with aligned BNs. Table S2: comparison of thermal conductivity of our work with other 3D printing and traditional methods. Figure S12: setup for the test of thermal control structures with 3D printed shapes. Figure S13: flame-retardant test of natural nacre. Figure S14: TGA tests of pure SI, BNs, and SI/55 wt% BNs and the [file 9840574.f1.zip › Supplemetal Figures/Figure S7.png]

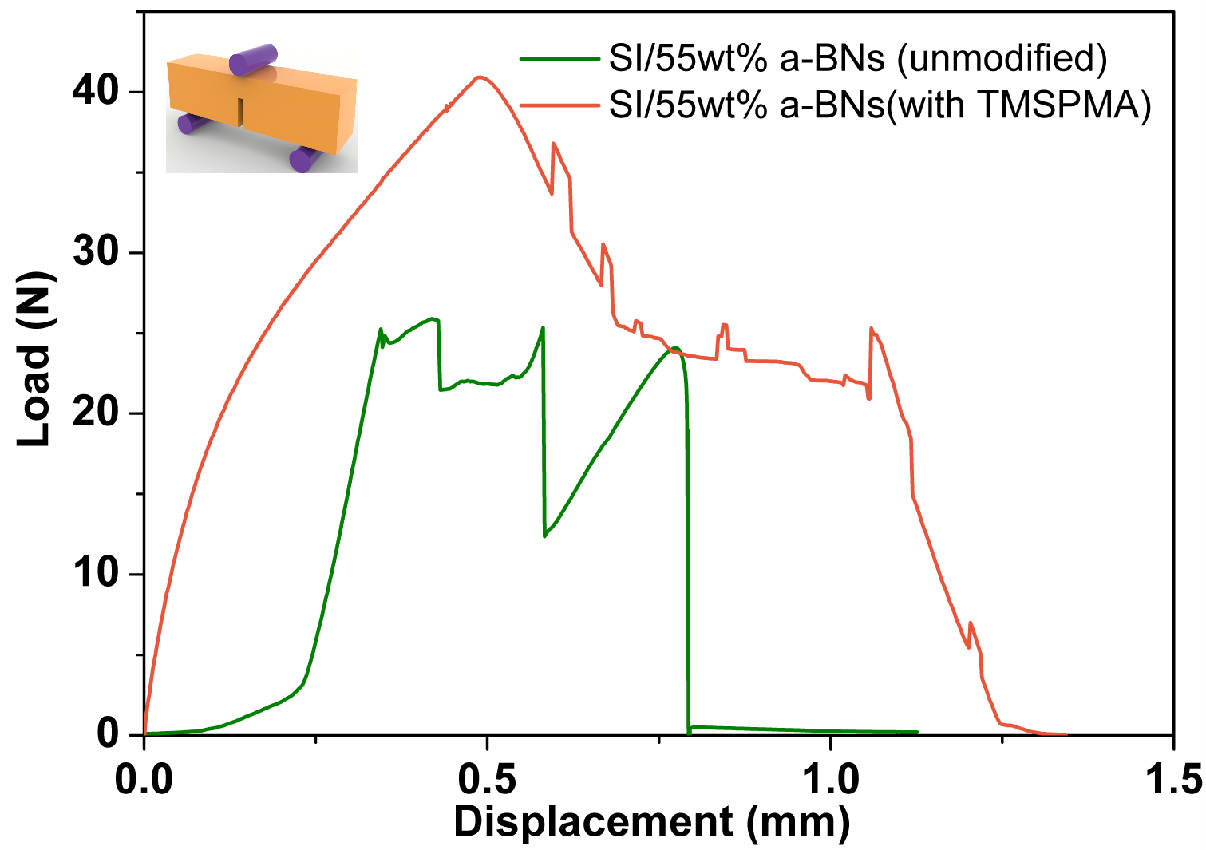

Supplement: Supplementary Materials — Table S1: comparison of mechanical protection property and shape complexity for boron nitride nanoplatelets (BNs) and nacre-inspired flame-retardant structures. Figure S1: SEM images of aligned BNs and magnified view of BNs. Figure S2: (a) representation of the surface modification procedure of BNs by 3-(trimethoxysilyl)propyl methacrylate (TMSPMA). (b) Schematic diagram shows the alignment of BNs in photocurable monomer and the covalent bonding between TMSPMA and photocurable monomer. Figure S3: FTIR spectrum of pure BNs and surface modified BNs by 3-(trimethoxysilyl)propyl methacrylate (TMSPMA). The diagram on the right shows the corresponding chemical bonding on the FTIR spectrum. Figure S4: SEM images of the original BNs (unmodified) and the TMSPMA-grafted BNs. Comparison of stress distribution during the sliding of adjacent BNs for the unmodified BNs and the TMSPMA-grafted BNs simulated by COMSOL Multiphysics. Figure S5: study of the efficiency of alignment of BNs with the gap between the doctor blade and the substrate, (a) 100 μm, (b) 300 μm,and (c) 500 μm. Figure S6: SEM images of SI/rBNs, SI/a-BNs with the unmodified BNs, and SI/a-BNs with the TMSPMA-grafted BNs. Figure S7: changes of cure depth with the fraction of BNs. Figure S8: comparison of 3-point-bending tests for 3D printed a-BNs with the unmodified BNs and the TMSPMA-grafted BNs. Figure S9: crack deflection, a-BN bridging, and pulling out for 3D printed nacre-inspired structures with TMSPMA grafted a-BNs. Figure S10: the standard three-point-bending tests were performed to study the flexural strength of the 3D-printed structures. Figure S11: compression test of the 3D printed nacre with aligned BNs. Table S2: comparison of thermal conductivity of our work with other 3D printing and traditional methods. Figure S12: setup for the test of thermal control structures with 3D printed shapes. Figure S13: flame-retardant test of natural nacre. Figure S14: TGA tests of pure SI, BNs, and SI/55 wt% BNs and the [file 9840574.f1.zip › Supplemetal Figures/Figure S8.png]

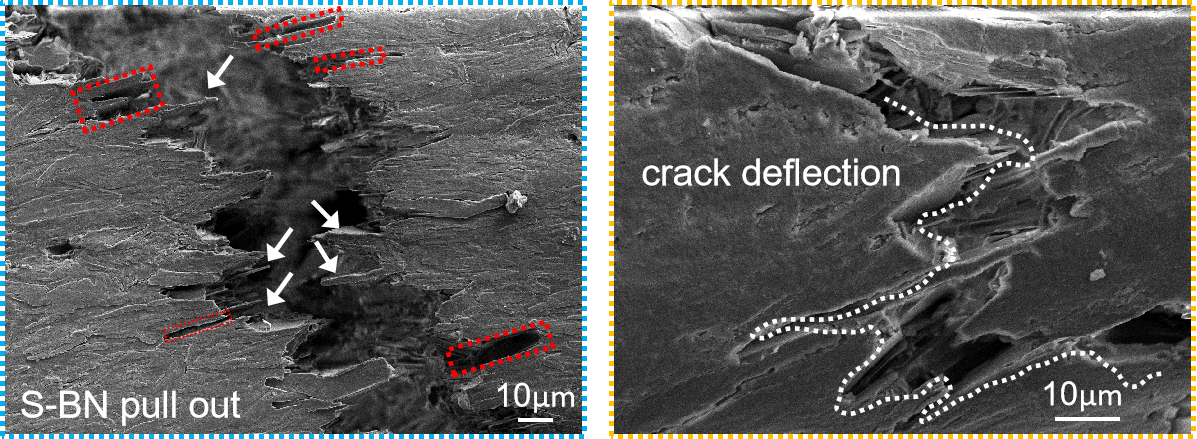

Supplement: Supplementary Materials — Table S1: comparison of mechanical protection property and shape complexity for boron nitride nanoplatelets (BNs) and nacre-inspired flame-retardant structures. Figure S1: SEM images of aligned BNs and magnified view of BNs. Figure S2: (a) representation of the surface modification procedure of BNs by 3-(trimethoxysilyl)propyl methacrylate (TMSPMA). (b) Schematic diagram shows the alignment of BNs in photocurable monomer and the covalent bonding between TMSPMA and photocurable monomer. Figure S3: FTIR spectrum of pure BNs and surface modified BNs by 3-(trimethoxysilyl)propyl methacrylate (TMSPMA). The diagram on the right shows the corresponding chemical bonding on the FTIR spectrum. Figure S4: SEM images of the original BNs (unmodified) and the TMSPMA-grafted BNs. Comparison of stress distribution during the sliding of adjacent BNs for the unmodified BNs and the TMSPMA-grafted BNs simulated by COMSOL Multiphysics. Figure S5: study of the efficiency of alignment of BNs with the gap between the doctor blade and the substrate, (a) 100 μm, (b) 300 μm,and (c) 500 μm. Figure S6: SEM images of SI/rBNs, SI/a-BNs with the unmodified BNs, and SI/a-BNs with the TMSPMA-grafted BNs. Figure S7: changes of cure depth with the fraction of BNs. Figure S8: comparison of 3-point-bending tests for 3D printed a-BNs with the unmodified BNs and the TMSPMA-grafted BNs. Figure S9: crack deflection, a-BN bridging, and pulling out for 3D printed nacre-inspired structures with TMSPMA grafted a-BNs. Figure S10: the standard three-point-bending tests were performed to study the flexural strength of the 3D-printed structures. Figure S11: compression test of the 3D printed nacre with aligned BNs. Table S2: comparison of thermal conductivity of our work with other 3D printing and traditional methods. Figure S12: setup for the test of thermal control structures with 3D printed shapes. Figure S13: flame-retardant test of natural nacre. Figure S14: TGA tests of pure SI, BNs, and SI/55 wt% BNs and the [file 9840574.f1.zip › Supplemetal Figures/Figure S9.png]
